# Cardiovascular complications are resolved by tuna protein hydrolysate supplementation in rats fed with a high-fat diet

**DOI:** 10.1038/s41598-023-39538-z

**Published:** 2023-07-28

**Authors:** Putcharawipa Maneesai, Jintanaporn Wattanathorn, Prapassorn Potue, Juthamas Khamseekaew, Siwayu Rattanakanokchai, Wipawee Thukham-Mee, Supaporn Muchimapura, Poungrat Pakdeechote

**Affiliations:** 1 Department of Physiology, Faculty of Medicine, Khon Kaen University, Khon Kaen, 40002 Thailand; 2 Research Institute for Human High Performance and Health Promotion, Khon Kaen University, Khon Kaen, 40002 Thailand; 3 Faculty of Veterinary Medicine, Khon Kaen University, Khon Kaen, 40002 Thailand

**Keywords:** Biochemistry, Physiology

## Abstract

This study is aimed to investigate whether tuna protein hydrolysate (TPH) supplementation could alleviate cardiovascular complications induced by a high-fat diet (HFD) in rats. Rats were fed a HFD for 16 weeks and given TPH (100 mg/kg, 300 mg/kg, or 500 mg/kg) or metformin (100 mg/kg) (n = 8) for the last four weeks. TPH had the following effects: resolved their impaired glucose tolerance, hyperglycemia, dyslipidemia, obesity, and hypertension (*p* < 0.05); alleviated left ventricular dysfunction and hypertrophy (*p* < 0.05), and vascular dysfunction and hypertrophy (*p* < 0.05); adipocyte hypertrophy; increases in circulating leptin and tumor necrosis factor (TNF-α) were mitigated (*p* < 0.05); increased renin-angiotensin system (RAS), oxidative stress, and decreased nitric oxide metabolites were modulated (*p* < 0.05). TPH restored the expression of angiotensin II receptor type 1 (AT1R)/NADPH oxidase 2 (NOX2), endothelial nitric oxide synthase (eNOS), nuclear factor erythroid 2-related factor (Nrf2)/heme oxygenase-1 (HO-1), and peroxisome proliferator-activated receptor γ (PPARγ)/the nuclear factor kappa B (NF-κB) protein in cardiovascular tissue (*p* < 0.05). In metabolic syndrome (MS) rats, metformin and TPH had comparable effects. In conclusion, TPH alleviated cardiovascular complications related to MS. It suppressed RAS, oxidative stress, and inflammation that were associated with modulation of AT1R/NOX2, eNOS, Nrf2/HO-1, and PPARγ/NF-κB expression.

## Introduction

Accumulating data indicate that metabolic abnormalities are notable causes of life-threatening diseases, especially cardiovascular disease^1^. Generally, signs of metabolic syndrome (MS), such as hyperglycemia, dyslipidemia, insulin resistance, central obesity, and hypertension, have been closely correlated with long-term excessive intake of a high-fat diet (HFD)^2^. Several rat models have demonstrated that feeding a HFD for eight weeks or more can generate metabolic abnormalities and cardiovascular complications^3–5^. Left ventricular (LV) contractile dysfunction and remodeling has also been shown in a rodent MS model^6,7^. These cardiac changes are suggested to be linked with high blood pressure and decreased insulin sensitivity^8^. Furthermore, impairment of endothelial function is observed in HFD fed rats and is closely related to hypertension^9^. In addition, the sympathetic nerves, which supply mesenteric arteries are activated leading to increasing vascular tone and blood pressure in rats with MS^9^. Changes in aortic structure, increases in wall thickness, and vascular smooth muscle cell proliferation and fibrosis, are also present in this animal model^10^. Increasing evidence indicates that factors including insulin resistance, modulation of the renin-angiotensin system (RAS), oxidative stress, and inflammation, participate in the underlying mechanisms of the cardiovascular abnormalities found in MS^11–13^.

Adipocyte hypertrophy is present in MS rats and is the major source of inflammatory cytokines and a peptide hormone, leptin; research reports that leptin can enhance RAS^14,15^. Hyperactivity of RAS contributes to metabolic disorder-related complications^16,17^. Moreover, an increase in RAS components including angiotensin-converting enzyme (ACE), angiotensin II (ang II), and angiotensin II receptor type 1 (AT_1_R) in local tissue and circulation were seen in diet-induced MS in rats^18^. It is well known that Ang II is the key RAS peptide mediating cardiovascular hypertrophy and failure^19^. RAS negatively affects the cardiovascular system via activation of the AngII/AT1R/NADPH oxidase cascade to generate superoxide^20,21^. Oxidative stress and inflammation contribute to the pathogenesis of cardiovascular change-related metabolic disorders^22,23^. Based on molecular protein expression, downregulation of nuclear factor erythroid 2-related factor (Nrf2) and heme oxygenase-1 (HO-1) protein associated with oxidative stress are observed in rats with metabolic disorders^24,25^. The regulation of inflammatory processes involves the ligand-activated transcription factor peroxisome proliferator-activated receptor γ (PPARγ) and the nuclear factor kappa B (NF-κB) signaling pathway. In HFD rats, a reduction of PPARγ protein expression and an enhancement of NF-κB expression related to inflammation were observed^26^.

Functional foods targeting cardiovascular illnesses linked to MS are now being studied. Fish protein hydrolysates are used as functional foods and are receiving increased attention. Several studies report the biological properties of protein hydrolysate-derived from marine fish. For example, protein hydrolysates isolated from mackerel exhibit a potential antioxidative peptide with high 2,2-diphenyl-1-picrylhydrazyl (DPPH) radical scavenging activity^27^. Antibacterial effects of the fish protein hydrolysate against Gram-positive and Gram-negative bacteria strains have been reported^28^. Anti-inflammatory effects of fish protein hydrolysate have been documented since supplementation of marine fish protein hydrolysates alleviate colitis-induced by dextran sodium sulfate in mice via suppressing the pro-inflammatory cytokines IL-6 and tumor necrosis factor (TNF)-α^29^. A previous report by Parolini et al. revealed that a salmon protein hydrolysate can reduced atherosclerotic lesions in apolipoprotein (apo) E(-/-) mice; this is associated with its anti-inflammatory effects of reducing circulating interleukin (IL)-1β , IL-6, and TNF-α^30^. Protein hydrolysate of tuna processing by-products show a ACE inhibitory activity, increased nitric oxide (NO) production, and suppression of norepinephrine-induced endothelin-1 secretion in human umbilical vein endothelial cells^31^. Nevertheless, only a few reports examine the effects of protein hydrolysates of tuna processing by-products on cardiovascular changes in MS rats. The purpose of this study was to explore whether tuna protein hydrolysate (TPH) supplementation could alleviate cardiovascular remodeling and dysfunction in rats fed a HFD.

## Results

### Compositions and amino acid profiles of TPH

The major component of THP was protein as shown in Table 1. Amino acid profiles are depicted in Table 2. The main content of amino acids in TPH was glutamic acid followed by aspartic acid and other 18 amino acids as shown in Table 2.

**Table 1:** Compositions of tuna protein hydrolysate.

Compositions	%
Protein	82.18 ± 2.78
Fat	0.32 ± 0.15
Ash	9.55 ± 1.05
Salt as NaCl	1.88 ± 1.24

**Table 2:** Amino acid profiles of tuna protein hydrolysate.

Amino acid (g/100 g)	
Glutamic acid	9.55 ± 0.90
Aspartic acid	6.84 ± 0.69
L-arginine	5.84 ± 0.36
Lysine	5.48 ± 0.80
Proline	5.08 ± 3.31
L-alanine	4.97 ± 0.35
Leucine	4.68 ± 2.12
Isoleucine	4.60 ± 3.37
Threonine	3.26 ± 0.33
Methionine	2.98 ± 1.86
Valine	2.79 ± 0.33
Phenylalanine	2.70 ± 0.18
Serine	2.66 ± 0.16
Histidine	2.43 ± 1.04
Tyrosine	2.17 ± 0.15
Glycine	1.48 ± 0.14
Tryptophan	0.37 ± 0.27
Cystine	0.34 ± 0.13
Hydroxyproline	0.18 ± 0.02
Hydroxylysine	0.04 ± 0.02

### Effects of TPH on body and organ weights

All rats fed a HFD plus 15% fructose solution for 16 weeks showed significant increases in BW, heart weight and EP pad compared to control rats (p < 0.05). TPH supplementation for weeks 12–16 at a dose of 300 mg/kg or 500 mg/kg, or metformin at a dose of 100 mg/kg significantly decreased BW and normalized the organ weight in MS rats compared to untreated-MS rats (p < 0:05). TPH (100 mg/kg) had no effect on BW and organ weight (Table 3).

**Table 3:** Effect of tuna protein hydrolysate on organ weight in HFD-induced MS rats.

Parameters	Control	MS	MS + T100	MS + T300	MS + T500	MS + Met100
BW (g)	642.25 ± 14.11	912.75 ± 41.47^a^	883.13 ± 39.60^a^	805.00 ± 23.36 ^a,b^	804.50 ± 11.10 ^a,b^	794.88 ± 17.59 ^a,b^
HW (g)	1.39 ± 0.03	1.87 ± 0.06^a^	1.78 ± 0.07^a^	1.63 ± 0.02 ^a,b^	1.66 ± 0.04 ^a,b^	1.65 ± 0.05 ^a,b^
LVW (g)	0.86 ± 0.03	1.27 ± 0.04^a^	1.17 ± 0.05^a^	1.04 ± 0.03^b^	1.03 ± 0.09^b^	1.05 ± 0.01^b^
EP pad weight (g)	9.66 ± 1.50	29.36 ± 1.57^a^	25.04 ± 1.38^a^	21.25 ± 1.91 ^a,b^	22.30 ± 1.10 ^a,b^	22.89 ± 1.06 ^a,b^
EP pad weight/BW (mg/g)	15.21 ± 1.71	33.76 ± 2.83^a^	29.93 ± 1.23^a^	25.73 ± 1.43 ^a,b^	26.58 ± 1.32 ^a,b^	26.31 ± 0.90 ^a,b^

Data are expressed as mean ± SEM.

*BW* body weight; *HW* heart weight; *LVW* left ventricular weight; *EP* epididymal fat; *MS* metabolic syndrome; *T100* tuna protein hydrolysate (100 mg/kg); *T300* tuna protein hydrolysate (300 mg/kg); *T500* tuna protein hydrolysate (500 mg/kg); *Met100* metformin (100 mg/kg).

^a^*p* < 0.05 vs. control.

^b^*p* < 0.05 vs. MS-only (n = 8/group).

### Effects of TPH oral glucose tolerance test (OGTT)

**Figure 1:**
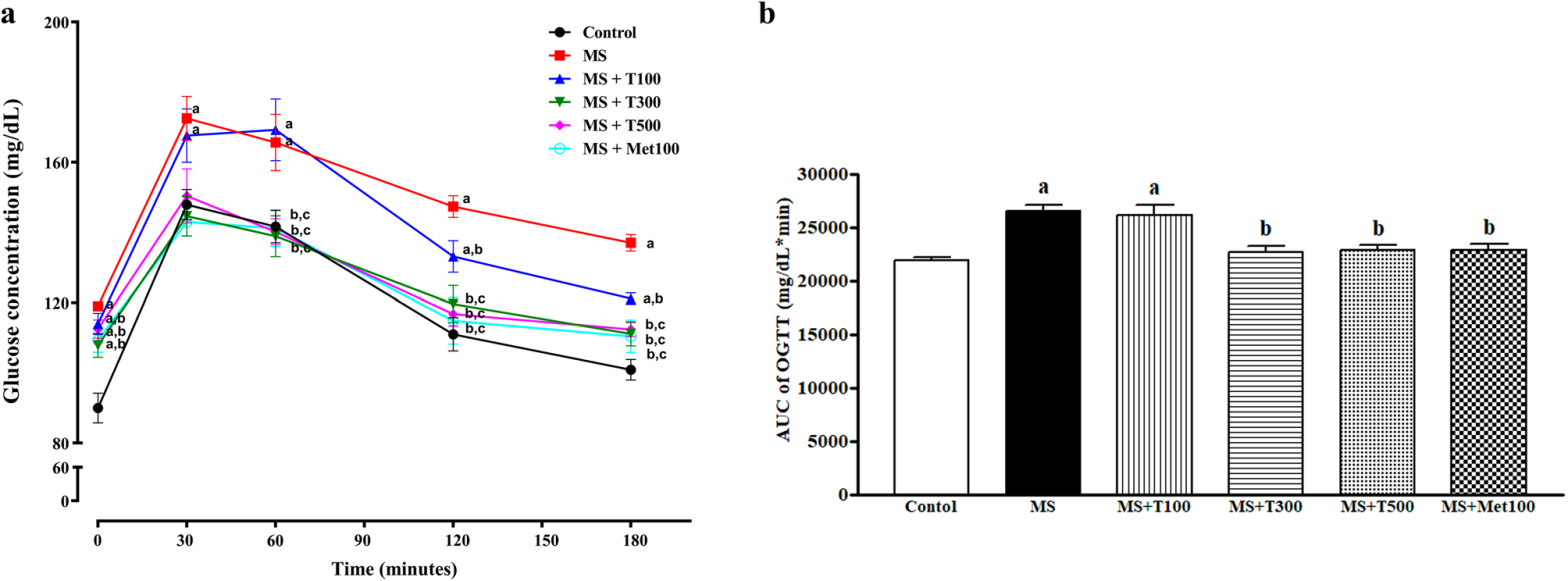
Changes of blood glucose concentrations in rats after receiving glucose (2 g/kg) for 180 min (**a**) and areas under the curves of oral glucose tolerances tests (OGTT) (**b**). Data are expressed as mean ± SEM. ^a^*p* < 0.05 vs control, ^b^*p* < 0.05 vs MS (n = 8/group). MS, metabolic syndrome; T100, tuna protein hydrolysate (100 mg/kg); T300, tuna protein hydrolysate (300 mg/kg); T500, tuna protein hydrolysate (500 mg/kg); Met100, metformin (100 mg/kg).

Fasting blood glucose (FBG) concentration at baseline glycemic level (T = 0 min) in MS rats was significantly higher than in controls (*p* < 0.05, Fig. 1a). Changes of plasma glucose (PG) concentrations during OGTT are presented in Fig. 1a. After 30 min of glucose administration, MS-only rats and MS rats + T100 showed the highest value of PG compared to control rats, while MS rats treated with T300, T500, or Met100 showed significantly decreased PG after 30 min of glucose administration when compared with MS rats (*p* < 0.05). PG levels of MS rats treated with T300, T500 or Met100 gradually reached baseline at 180 min. Figure 1b depicts the area under the curve (AUC) of OGTT of all MS rats; it was significantly higher than in control rats. Treatment with TPH (300 or 500 mg/kg) or metformin restored the AUC of OGTT in MS rats (*p* < 0.05). However, the AUC of OGTT of MS rats treated with TPH (100 mg/kg) was similar to the MS group.

### Effects of TPH on metabolic profiles, leptin, TNF α, and liver enzymes in rats

All MS rats showed dyslipidemia, high levels of total cholesterol (TC) and triglyceride (TG), as well as low levels of high-density lipoprotein cholesterol (HDL-c) in plasma compared to normal controls (*p* < 0.05). TPH or metformin alleviated dyslipidemia induced by the HFD. High concentrations of plasma leptin and TNF-α were found in MS rats and these were attenuated by TPH or metformin supplementation (*p* < 0.05; Data shown in Table 4).

**Table 4:** Metabolic profiles, leptin, and TNF-α levels in rats.

Parameters	Control	MS	MS + T100	MS + T300	MS + T500	MS + Met100
FBG (mg/mL)	88.00 ± 2.57	119.00 ± 1.07 ^a^	114.00 ± 3.00^a^	107.30 ± 2.97 ^a,b^	109.67 ± 2.25 ^a,b^	107.33 ± 3.33 ^a,b^
TC (mmol/L)	0.38 ± 0.03	0.63 ± 0.04 ^a^	0.45 ± 0.04^b^	0.38 ± 0.03 ^b^	0.38 ± 0.03^b^	0.40 ± 0.02^b^
TG (mmol/L)	0.14 ± 0.01	0.38 ± 0.06^a^	0.22 ± 0.03^a^	0.14 ± 0.01^b^	0.15 ± 0.01^b^	0.17 ± 0.01^b^
HDL-c (mmol/L)	0.57 ± 0.08	0.22 ± 0.02^a^	0.35 ± 0.04 ^a,b^	0.46 ± 0.04^b^	0.45 ± 0.03^b^	0.47 ± 0.06^b^
leptin (ng/mL)	1.15 ± 0.02	5.07 ± 0.08 ^a^	2.94 ± 0.03 ^a,b^	1.25 ± 0.03^b^	1.50 ± 0.03^b^	1.49 ± 0.03 ^b^
TNF-α (pg/mL)	109.56 ± 28.84	715.43 ± 74.81^a^	537.33 ± 85.69^a^	189.90 ± 36.41 ^b,c^	131.62 ± 17.05 ^b,c^	160.89 ± 22.60 ^b,c^

Data are expressed as mean ± SEM.

*FBG* fasting blood glucose; *TC* total cholesterol; *TG* total triglyceride; *HDL-c* high density lipoprotein-cholesterol; *TNF-α* tumor necrosis factor alpha; *MS* metabolic syndrome; *T100* tuna protein hydrolysate (100 mg/kg); *T300* tuna protein hydrolysate (300 mg/kg); *T500* tuna protein hydrolysate (500 mg/kg); *Met100* metformin (100 mg/kg).

^a^*p* < 0.05 vs. control.

^b^*p* < 0.05 vs. MS (n = 8/group).

### Effects of TPH on hemodynamic profiles

**Figure 2:**
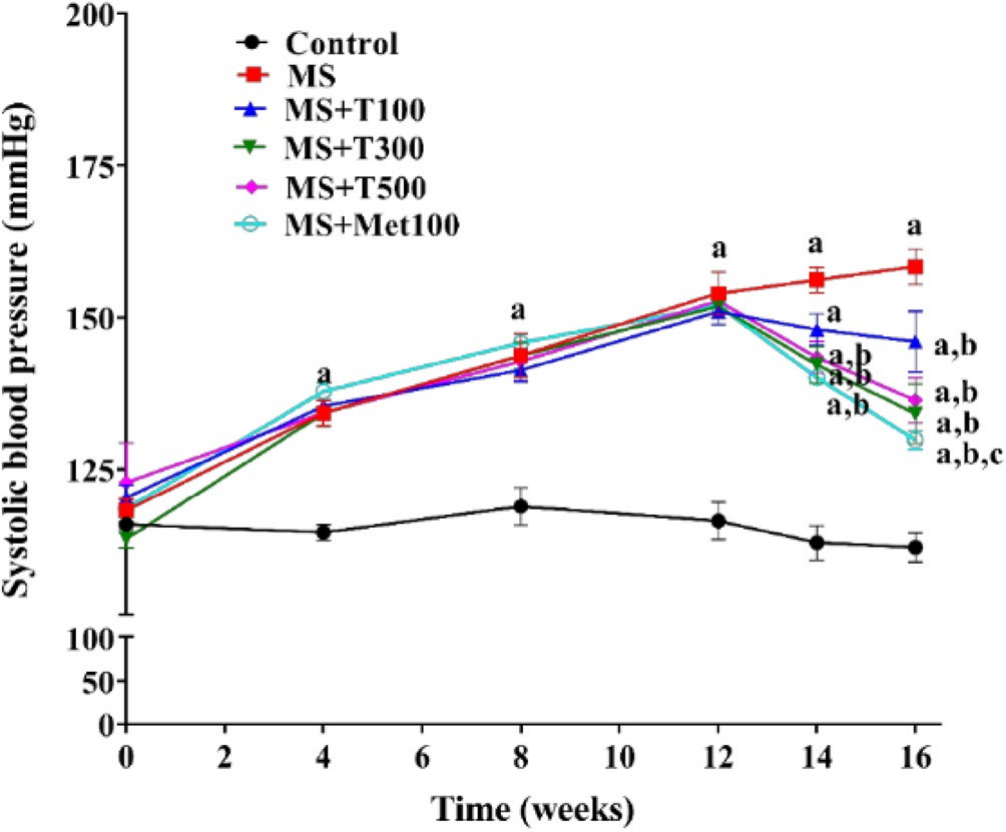
Systolic blood pressure changes over the course of the 16-week study. Data are expressed as mean ± SEM. ^a^*p* < 0.05 vs control, ^b^*p* < 0.05 vs MS, ^c^*p* < 0.05 vs MS + T100 (n = 8/group). MS, metabolic syndrome; T100, tuna protein hydrolysate (100 mg/kg); T300, tuna protein hydrolysate (300 mg/kg); T500, tuna protein hydrolysate (500 mg/kg); Met100, metformin (100 mg/kg).

Systolic blood pressure (SP) of all MS rats dramatically increased after a four-week HFD (*p* < 0.05). SP gradually increased over 16 weeks to 158.39 ± 2.98 mmHg in MS rats while control rats had a normal SP at 115.90 ± 0.64 mmHg. TPH (300 or 500 mg/kg) or metformin supplementation during the final four weeks significantly reduced SP compared to MS rats (139.78 ± 5.41, 138.84 ± 4.11, and 129.40 ± 1.70 mmHg, respectively, *p* < 0.05; Fig. 2). Meanwhile TPH at a dose of 100 or 500 mg/kg had no effect on SP. These SP values were consistent with the intra-arterial pressure obtained following 16 weeks of research, since high values of SP, diastolic blood pressure (DP), mean arterial pressure (MAP), pulse pressure (PP), and heart rate (HR) were observed in all MS rats compared to controls (*p* < 0.05). Daily treatment with TPH or metformin for four weeks significantly improved hemodynamic parameters in MS rats compared to controls (*p* < 0.05, Table 5).

**Table 5:** Hemodynamic profiles in all rats.

Parameters	Control	MS	MS + T100	MS + T300	MS + T500	MS + Met100
SP (mmHg)	110.51 ± 2.86	158.22 ± 1.54 ^a^	131.84 ± 5.26 ^a,b^	121.37 ± 2.72 ^b^	126.78 ± 1.60 ^b^	122.46 ± 4.17 ^b^
DP (mmHg)	71.06 ± 1.48	103.71 ± 2.05 ^a^	78.64 ± 2.57 ^a,b^	71.71 ± 3.81 ^b^	73.41 ± 2.68 ^b^	76.43 ± 3.61 ^b^
MAP (mmHg)	82.70 ± 2.79	122.50 ± 1.30 ^a^	96.48 ± 3.42 ^a,b^	85.71 ± 4.72 ^b^	91.27 ± 1.43 ^b^	93.15 ± 3.59 ^b^
PP (mmHg)	39.41 ± 1.96	56.13 ± 3.08 ^a^	53.09 ± 3.49^a^	50.43 ± 2.32	49.37 ± 1.94	45.55 ± 2.62
HR (beats/min)	398.29 ± 26.76	507.03 ± 14.45 ^a^	462.81 ± 21.61	430.71 ± 28.20	416.90 ± 23.25	414.57 ± 23.37

Data are expressed as mean ± SEM.

*SP* systolic blood pressure; *DP* diastolic blood pressure; *MAP* mean arterial pressure; *PP* pulse pressure; *HR* heart rate; *MS* metabolic syndrome; *T100* tuna protein hydrolysate (100 mg/kg); *T300* tuna protein hydrolysate (300 mg/kg); *T500* tuna protein hydrolysate (500 mg/kg); *Met100* metformin (100 mg/kg).

^a^*p* < 0.05 vs control.

^b^*p* < 0.05 vs. MS (n = 8/group).

### Effect of TPH on left ventricular (LV) function and structure

**Figure 3:**
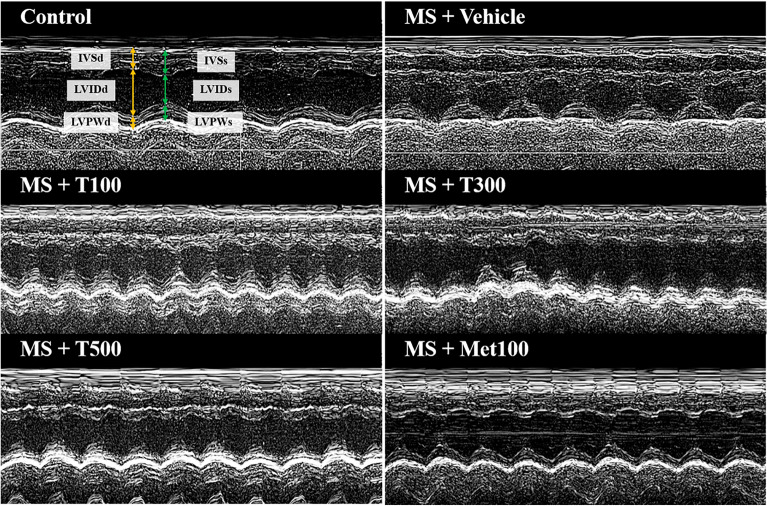
Example of echocardiographic tracings in each group. MS, metabolic syndrome; T100, tuna protein hydrolysate (100 mg/kg); T300, tuna protein hydrolysate (300 mg/kg); T500, tuna protein hydrolysate (500 mg/kg); Met100, metformin (100 mg/kg).

An example echocardiographic tracing for each group is shown in Fig. 3. MS rats had impaired LV performance as evidenced by decreased end diastolic volume (EDV), stroke volume (SV), LV fraction shortening (%FS), and ejection fraction (EF) compared to controls (*p* < 0.05). In addition, LV enlargement (lowered internal dimension at end-diastole (LVIDd) but increased LV internal dimension at end-systole (LVPWd)) was seen in MS rats compared to controls (*p* < 0.05). With TPH or metformin supplementation, LV dysfunction and hypertrophy were restored in MS rats (*p* < 0.05; Table 6).

**Table 6:** Effect of TPH on LV function and structure in MS rats.

Parameters	Control	MS	MS + T100	MS + T300	MS + T500	MS + Met100
IVSd (cm)	0.186 ± 0.00	0.188 ± 0.01	0.188 ± 0.01	0.191 ± 0.01	0.185 ± 0.01	0.193 ± 0.01
IVSs (cm)	0.294 ± 0.01	0.276 ± 0.01	0.297 ± 0.01	0.294 ± 0.02	0.297 ± 0.01	0.292 ± 0.01
LVIDd (cm)	0.829 ± 0.02	0.706 ± 0.03^a^	0.770 ± 0.01	0.823 ± 0.03^b^	0.832 ± 0.02^b^	0.865 ± 0.03^b^
LVIDs (cm)	0.509 ± 0.01	0.471 ± 0.03	0.482 ± 0.03	0.456 ± 0.02	0.478 ± 0.03	0.536 ± 0.01
LVPWd (cm)	0.206 ± 0.01	0.241 ± 0.01^a^	0.204 ± 0.00^b^	0.206 ± 0.01^b^	0.203 ± 0.01^b^	0.204 ± 0.01^b^
LVPWs (cm)	0.29 ± 0.01	0.29 ± 0.01	0.309 ± 0.01	0.30 ± 0.02	0.29 ± 0.00	0.30 ± 0.01
EDV (mL)	1.209 ± 0.07	0.819 ± 0.09^a^	1.145 ± 0.11	1.29 ± 0.08^b^	1.32 ± 0.08^b^	1.26 ± 0.14^b^
ESV (mL)	0.325 ± 0.02	0.329 ± 0.02	0.291 ± 0.06	0.332 ± 0.02	0.330 ± 0.03	0.323 ± 0.04
EF (%)	75.03 ± 1.06	64.45 ± 2.24^a^	77.08 ± 2.09^b^	77.49 ± 2.40^b^	78.22 ± 1.69^b^	74.92 ± 0.78^b^
SV (mL)	0.884 ± 0.06	0.493 ± 1.24^a^	0.896 ± 0.08^b^	0.958 ± 0.04^b^	0.974 ± 0.06^b^	0.953 ± 0.10^b^
FS (%)	38.29 ± 1.31	31.08 ± 1.24^a^	38.92 ± 1.44^b^	41.71 ± 2.34^b^	42.38 ± 1.58^b^	39.63 ± 0.54^b^

Data are expressed as mean ± SEM.

*IVSd* interventricular septum at diastole; *IVSs* interventricular septum at systole; *LVIDd* left ventricular internal dimension at end-diastole; *LVIDs* left ventricular internal dimension at end-systole; *LVPWd* left ventricular posterior wall thickness at diastole; *LVPWs* left ventricular posterior wall thickness at systole; *EDV* end diastolic volume; *ESV* end systolic volume; *SV* stroke volume; *EF* ejection fraction; *FS* fractional shortening; *MS* metabolic syndrome; *T100* tuna protein hydrolysate (100 mg/kg); *T300* tuna protein hydrolysate (300 mg/kg); *T500* tuna protein hydrolysate (500 mg/kg); *Met100* metformin (100 mg/kg).

^a^*p* < 0.05 vs control.

^b^*p* < 0.05 vs. MS (n = 7/group).

### Effect of TPH on LV morphology

**Figure 4:**
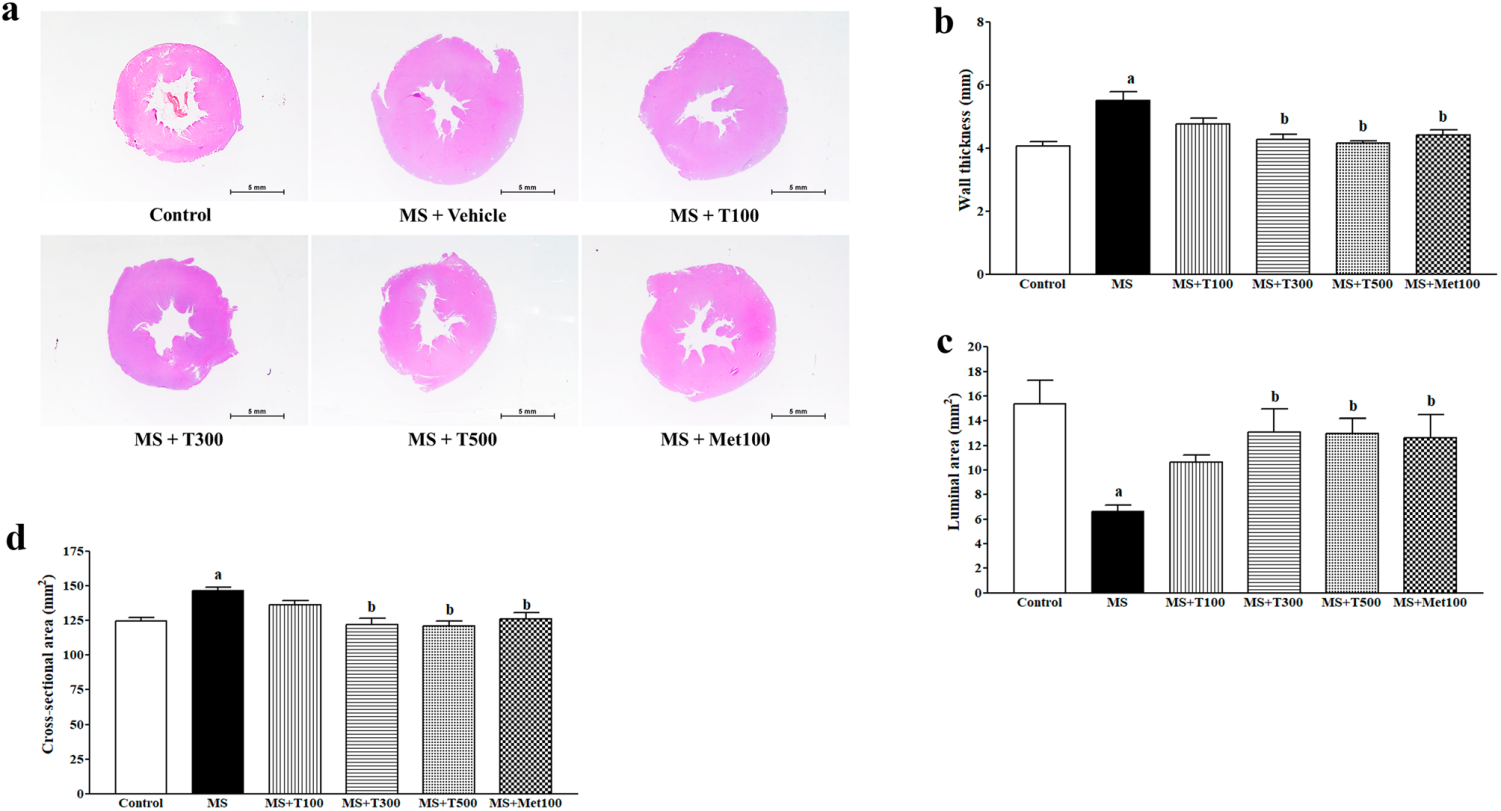
Example images of the cardiac morphological changes in each group; stained with H&E (**a**) (Scale bar = 5 mm). Quantitative data of wall thickness (**b**), luminal area (**c**), and cross-sectional area (**d**) of left ventricle in MS rats. MS, metabolic syndrome; T100, tuna protein hydrolysate (100 mg/kg); T300, tuna protein hydrolysate (300 mg/kg); T500, tuna protein hydrolysate (500 mg/kg); Met100, metformin (100 mg/kg).

Figure 4a depicts cardiac morphological changes in each group. Hematoxylin and eosin (H&E) staining shows increased LV wall thickness (Fig. 4b) and CSA (Fig. 4d) but reduced luminal area (Fig. 4c) in all MS rats compared to controls (*p* < 0.05). The cardiac hypertrophy induced by a HFD was alleviated by TPH (300 or 500 mg/kg) or metformin (*p* < 0.05). TPH (100 mg/kg) did not alter cardiac morphology in MS rats.

### Effect of TPH on vascular function

**Figure 5:**
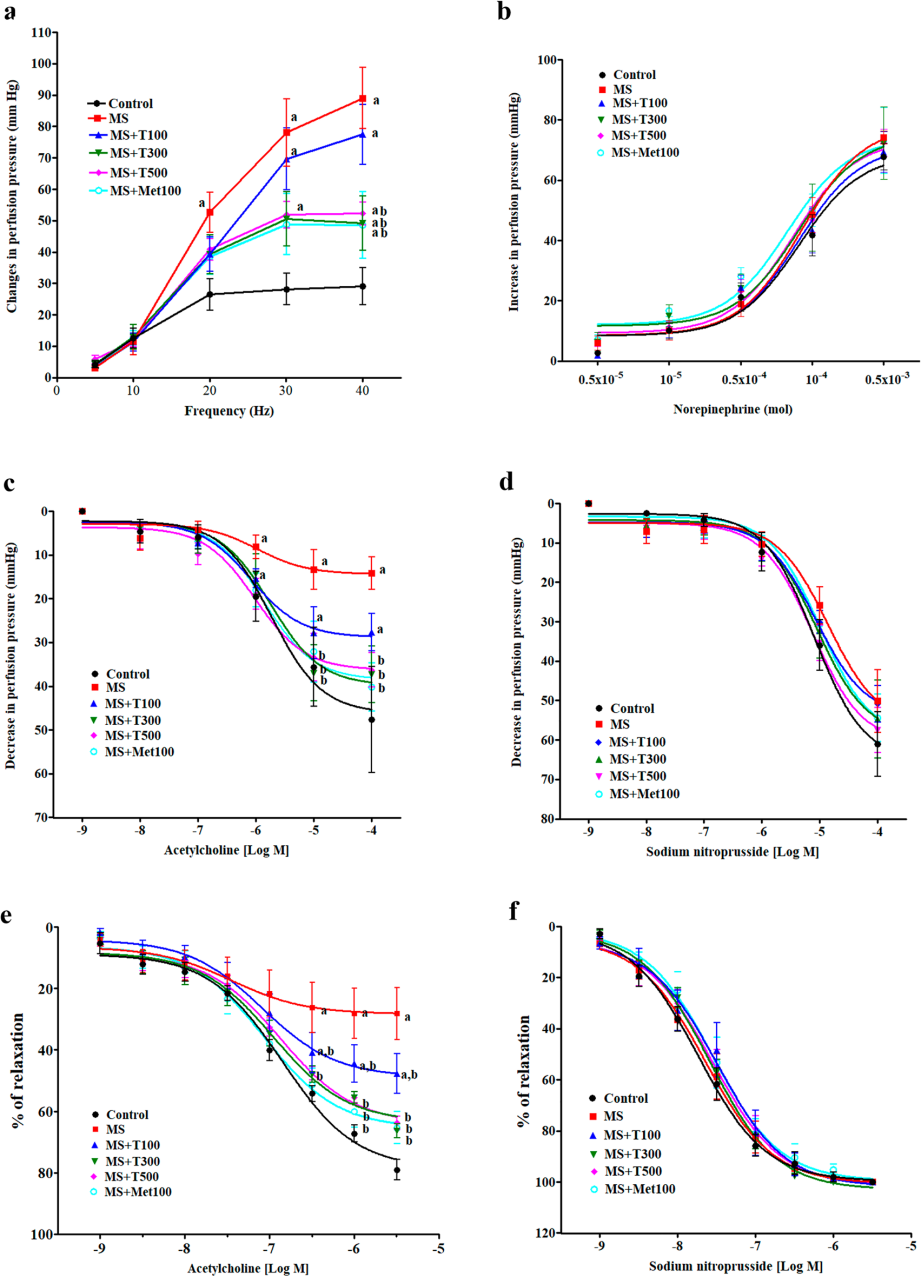
Effect of TPH on vascular responses to EFS (**a**), exogenous NE (**b**) ACh (**c**) SNP (**d**) in mesenteric vascular beds, and vascular responses to ACh (**e**) and SNP (**f**) in aortic rings. Data are expressed as mean ± SEM. ^a^*p* < 0.05 vs control, ^b^*p* < 0.05 vs MS, ^c^*p* < 0.05 vs MS + T100 (n = 7/group). MS, metabolic syndrome; T100, tuna protein hydrolysate (100 mg/kg); T300, tuna protein hydrolysate (300 mg/kg); T500, tuna protein hydrolysate (500 mg/kg); Met100, metformin (100 mg/kg).

The contractile responses to electrical field stimulation (EFS) were larger in those preparations isolated from MS rats than controls (*p* < 0.05; Fig. 5a). TPH (300 or 500 mg/kg) or metformin reduced sympathetic nerve mediated contractile responses in MS rats compared to untreated rats (*p* < 0.05). The contractile response to exogenous norepinephrine (NE) did not differ significantly between the groups (Fig. 5b). MS rats had reduced relaxation responses to acetylcholine (ACh), and endothelium-dependent vasorelaxation in both mesenteric vascular beds and aortic rings (*p* < 0.05; Fig. 5c,e). TPH (300 or 500 mg/kg) or metformin significantly improved endothelial function by restoring vasodilation induced by the ACh in both preparations (*p* < 0.05). The vascular responses to sodium nitroprusside (SNP) were not significantly different between groups (Fig. 5d,f).

### Effect of TPH on aortic hypertrophy

**Figure 6:**
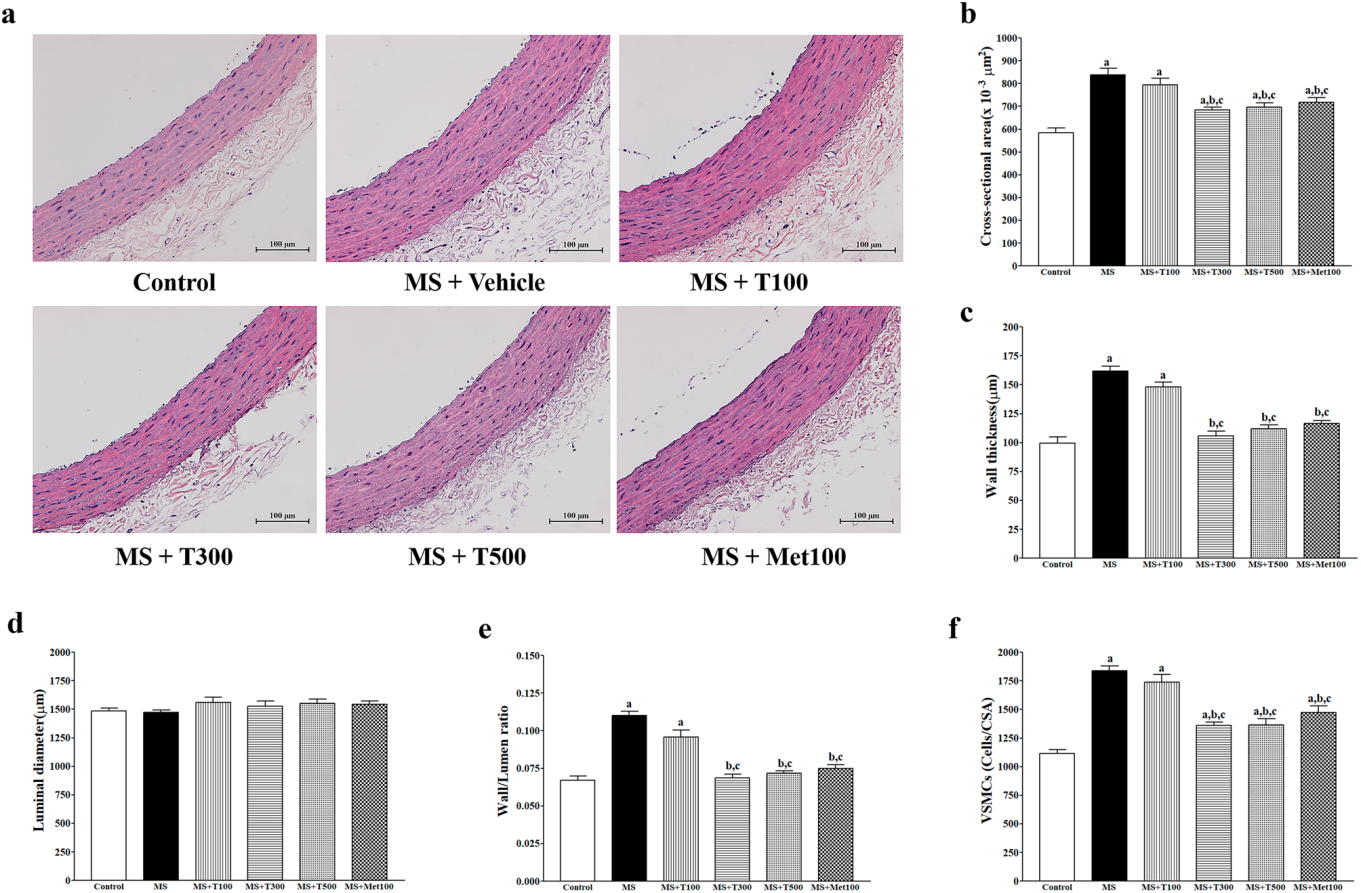
Effect of TPH on aortic hypertrophy. Representative images of aorta; stained by H&E (**a**) (Scale bar = 100 µm). Quantitative data of cross-sectional area (**b**), wall thickness (**c**), luminal diameter (**d**), wall/lumen ratio (**e**), and vascular smooth muscle cell numbers (f) in thoracic aorta. Data are expressed as mean ± SEM. ^a^*p* < 0.05 vs control, ^b^*p* < 0.05 vs MS, ^c^*p* < 0.05 vs MS + T100 (n = 7/group). MS, metabolic syndrome; T100, tuna protein hydrolysate (100 mg/kg); T300, tuna protein hydrolysate (300 mg/kg); T500, tuna protein hydrolysate (500 mg/kg); Met100, metformin (100 mg/kg).

Representative pictures of vascular staining with H&E are depicted in Fig. 6a. Aortic hypertrophy was noted, it was indicated by increasing cross sectional area (CSA) (Fig. 6b), wall thickness (Fig. 6c), wall/lumen ratios (Fig. 6d), wall per lumen ratio (Fig. 6e), and vascular smooth muscle cell (VSMC; Fig. 6f) number in the aortas of MS rats compared to the control group (*p* < 0.05). TPH (300 or 500 mg/kg) or metformin alleviated aortic hypertrophy in MS-treated rats (*p* < 0.05). However, the luminal diameters did not differ between groups, as shown in Fig. 6d.

### Effect of TPH on epididymal fat morphology

**Figure 7:**
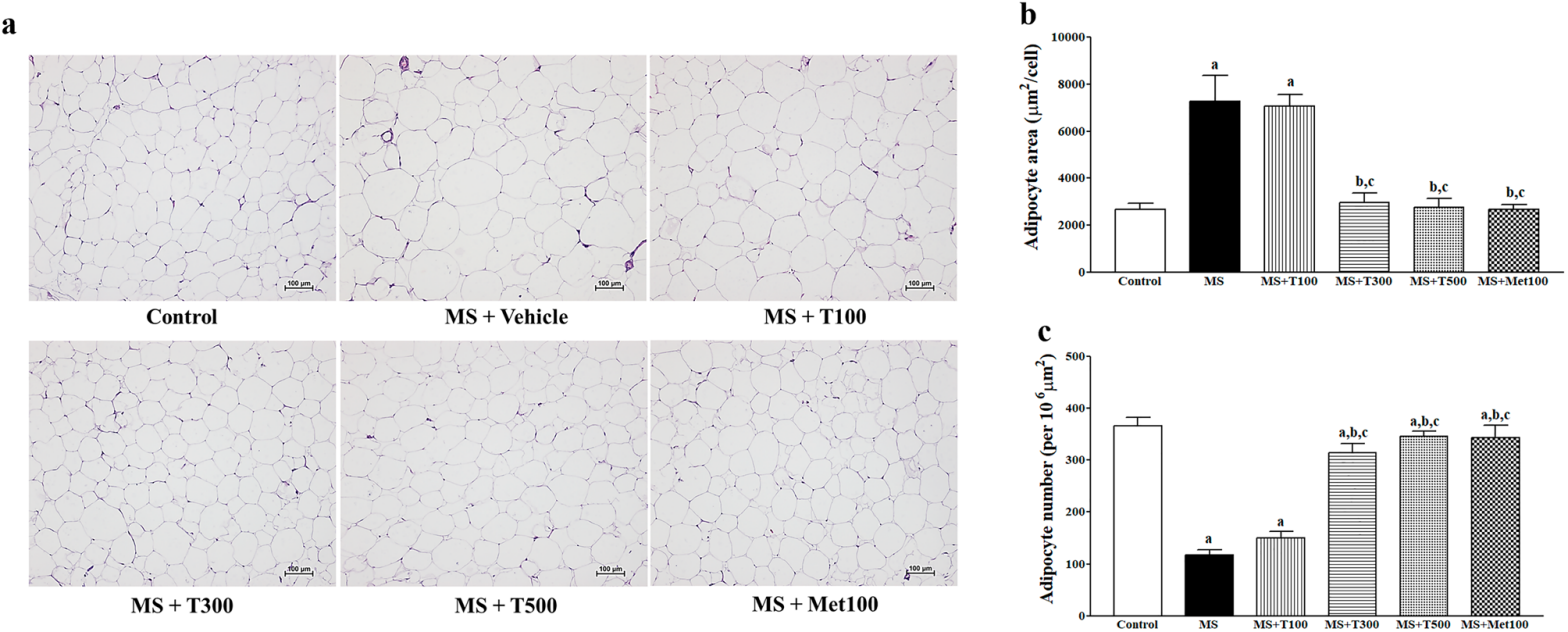
Example pictures of epididymal fat sections (**a**) (magnifcation × 10, scale bar = 100 μm) stained with H&E. Quantitative data of areas of adipocyte cells (**b**) and number of adipocytes per unit area (**c**). Data are expressed as mean ± SEM. ^a^*p* < 0.05 vs control, ^b^*p* < 0.05 vs. MS, ^c^*p* < 0.05 vs MS + LM50 (n = 7/group). MS, metabolic syndrome; T100, tuna protein hydrolysate (100 mg/kg); T300, tuna protein hydrolysate (300 mg/kg); T500, tuna protein hydrolysate (500 mg/kg); Met100, metformin (100 mg/kg).

Figure 7 shows a representative view of the epididymal fat pad slice. An increase in cell size of adipocytes from epididymal fat pads (Fig. 7a) accompanied by a reduction of cell number per area (Fig. 7b) were revealed in MS rats. TPH (300 or 500 mg/kg) or metformin supplement reversed the size and number of adipocytes compared to controls (*p* < 0.05).

### Effect of TPH on RAS

**Figure 8:**
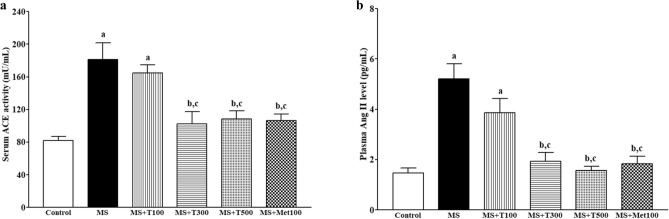
Effect of tuna protein hydrolysate on serum ACE activity (**a**) and plasma Ang II concentration (**b**). Data are expressed as mean ± SEM. ^a^*p* < 0.05 vs control, ^b^*p* < 0.05 vs MS, ^c^*p* < 0.05 vs MS + T100 (n = 7/group). ACE, angiotensin converting enzyme; Ang II, angiotensin II. MS, metabolic syndrome; T100, tuna protein hydrolysate (100 mg/kg); T300, tuna protein hydrolysate (300 mg/kg); T500, tuna protein hydrolysate (500 mg/kg); Met100, metformin (100 mg/kg).

RAS was observed to be activated in MS animals as evidenced by increases in plasma Ang II levels and serum ACE activity (Fig. 8a,b) compared to the control group. (*p* < 0.05). Treatment with TPH (300 or 500 mg/kg) or metformin ameliorated RAS activation by reducing ACE activity and Ang II concentration compared to untreated rats (*p* < 0.05).

### Effect of TPH on oxidative stress markers

Oxidative stress was observed in all MS rats. This was corroborated by increasing superoxide (O_2_^•−^) generation in the aorta and heart, higher malondialdehyde (MDA) levels in the heart, and the aorta, but decreasing catalase (CAT) activity in the heart and the aorta when compared to controls (*p* < 0.05). TPH or metformin decreased oxidative stress biomarkers and raised CAT activity in tissue compared to MS rats (*p* < 0.05; Table 7).

**Table 7:** Effect of TPH on oxidative stress parameters.

Parameters	Control	MS	MS + T100	MS + T300	MS + T500	MS + Met100
O_2_^•−^ production in heart (Count/mg dry wt/min)	68.66 ± 13.64	147.56 ± 19.70^a^	95.66 ± 3.04^b^	64.96 ± 3.34^b^	68.82 ± 9.58^b^	71.74 ± 9.62^b^
O_2_^•−^ production in aorta (Count/mg dry wt/min)	43.02 ± 5.62	77.86 ± 9.36^a^	69.80 ± 6.38^a^	38.93 ± 2.22 ^b,c^	42.81 ± 5.61 ^b,c^	44.02 ± 4.44 ^b,c^
MDA in heart (µmol/g)	4.00 ± 0.19	9.36 ± 0.33 ^a^	7.23 ± 0.26 ^a,b^	5.40 ± 0.26 ^b,c^	4.87 ± 0.50 ^b,c^	5.20 ± 0.36 ^b,c^
MDA in aorta (µmol/g)	1.30 ± 0.10	3.73 ± 0.24 ^a^	2.39 ± 0.15 ^a,b^	1.73 ± 0.12^b^	1.90 ± 0.21^b^	2.04 ± 0.25^b^
CAT in heart (U/mg)	9.16 ± 0.38	3.72 ± 0.30^a^	4.90 ± 0.27^a^	7.69 ± 0.29 ^b,c^	7.95 ± 0.49 ^b,c^	7.96 ± 0.50 ^b,c^
CAT in aorta (U/mg)	8.10 ± 0.37	4.74 ± 0.28^a^	5.78 ± 0.41^a^	7.36 ± 0.39 ^b,c^	7.51 ± 0.49 ^b,c^	7.92 ± 0.58 ^b,c^

Data are expressed as mean ± SEM.

*O*_*2*_^*•−*^ superoxide anion; *MDA* malondialdehyde; *CAT* catalase; *MS* metabolic syndrome; *T100* tuna protein hydrolysate (100 mg/kg); *T300* tuna protein hydrolysate (300 mg/kg); *T500* tuna protein hydrolysate (500 mg/kg); *Met100* metformin (100 mg/kg).

^a^p < 0.05 vs. control.

^b^p < 0.05 vs. MS.

^c^p < 0.05 vs. MS + T100 (n = 7/group).

### Effect of TPH on AT1R and gp91phox protein expressions in cardiac tissue

**Figure 9:**
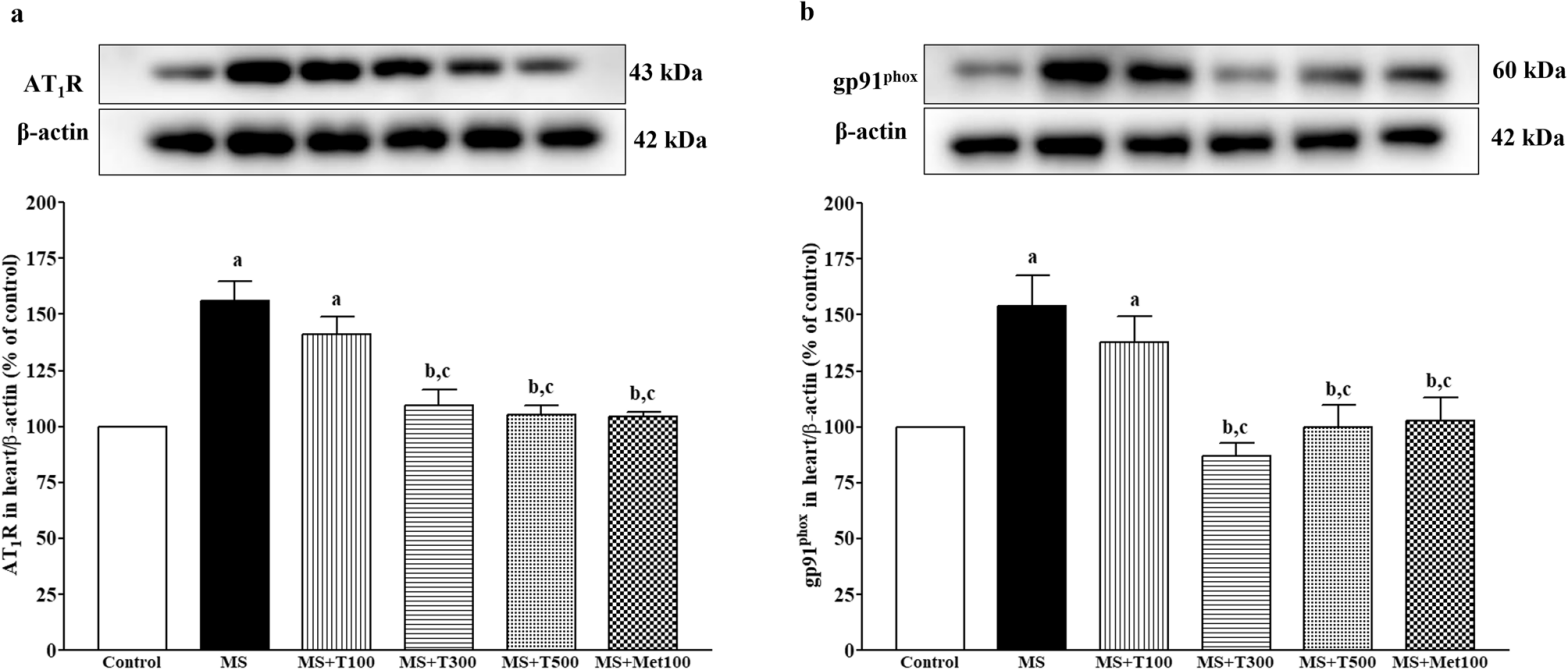
Effect of TPH on protein expression of AT_1_R (**a**) and gp91^phox^ (**b**) in cardiac tissue. Data are expressed as mean ± SEM. ^a^*p* < 0.05 vs control, ^b^*p* < 0.05 vs MS, ^c^*p* < 0.05 vs MS + T100 (n = 4/group). AT1R, angiotensin II receptor type I; gp91^phox^, NADPH oxidase subunit 2; angiotensin II receptor type I; MS, metabolic syndrome; T100, tuna protein hydrolysate (100 mg/kg); T300, tuna protein hydrolysate (300 mg/kg); T500, tuna protein hydrolysate (500 mg/kg); Met100, metformin (100 mg/kg). Original blots are presented in Supplementary Fig. 9.

Western blot analysis showed upregulation of AT_1_R and gp91^phox^ protein expression in cardiac tissue of all MS rats when compared to controls (p < 0.05). In comparison to, TPH (300 or 500 mg/kg) or metformin administration decreased the expression of the AT_1_R and gp91^phox^ proteins (*p* < 0.05, Fig. 9).

### Effect of TPH on nitric oxide metabolites (NOx) and endothelial nitic oxide synthase (eNOS) protein expression in aortic tissue

**Figure 10:**
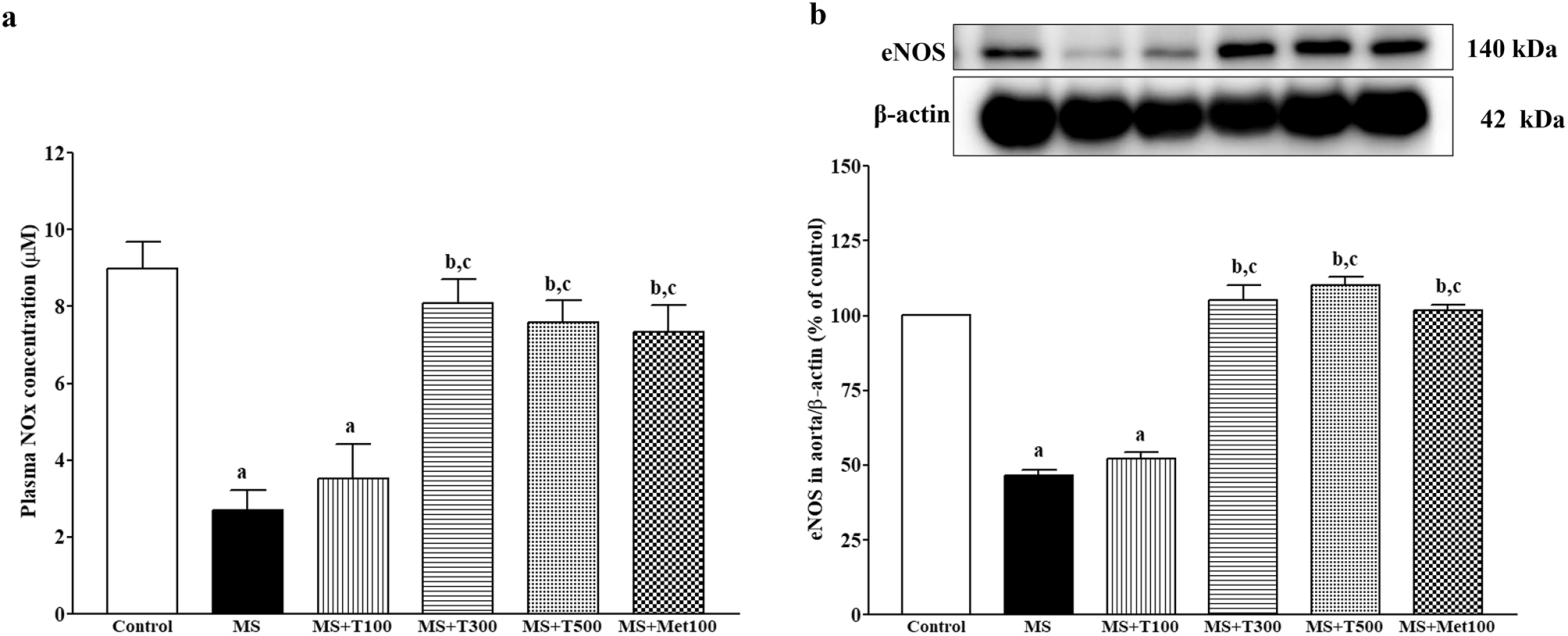
Effect of TPH on plasma NOx (n = 7/group) (**a**) and eNOS protein expression in aorta (n = 4) (**b**). Data are expressed as mean ± SEM. ^a^*p* < 0.05 vs control, ^b^*p* < 0.05 vs MS, ^c^*p* < 0.05 vs MS + T100. NOx, nitric oxide metabolites; eNOS, endothelial nitric oxide synthase; MS, metabolic syndrome; T100, tuna protein hydrolysate (100 mg/kg); T300, tuna protein hydrolysate (300 mg/kg); T500, tuna protein hydrolysate (500 mg/kg); Met100, metformin (100 mg/kg). Original blots are presented in Supplementary Fig. 10.

eNOS protein expression was decreased in the aortic tissue of MS rats compared to control rats (Fig. 10a). The reduction of eNOS protein expression was accompanied by low levels of plasma NOx in MS rats (*p* < 0.05, Fig. 10b). TPH (300 or 500 mg/kg) or metformin administration restored both eNOS expression and circulating NOx concentration in MS-treated rats compared to MS rats (*p* < 0.05).

### Effect of TPH on HO-1, Nrf-2, PPARγ, and phosphorylated nuclear factor kappa B (p-NF-κB) protein expression in aortic tissue

**Figure 11:**
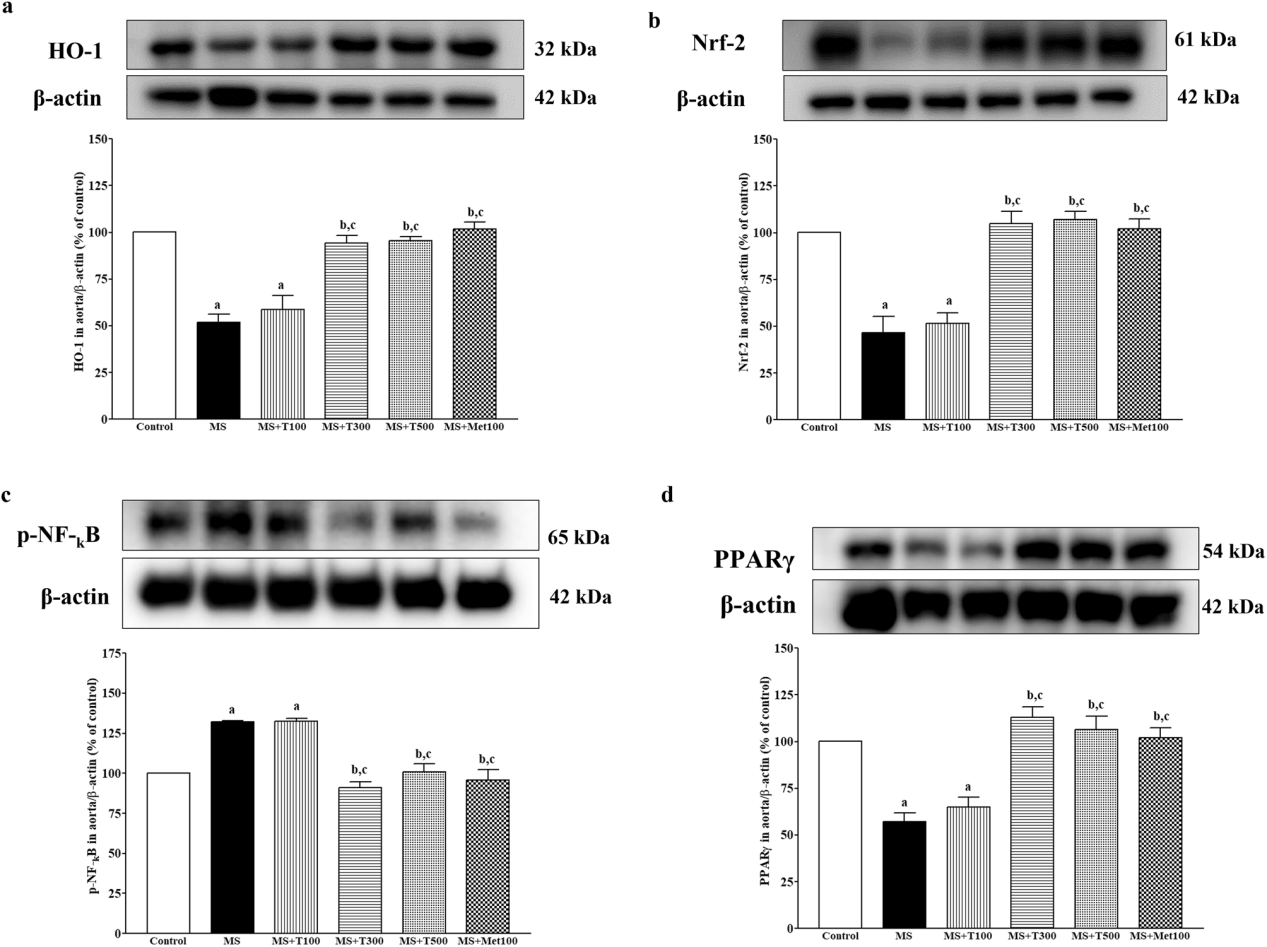
Effect of TPH on the expression of HO-1 (**a**), Nrf-2 (**b**), p-NF-kB (**c**), and PPARγ (d) in aortic tissue. Data are expressed as mean ± SEM. ^a^*p* < 0.05 vs control, ^b^*p* < 0.05 vs MS, ^c^*p* < 0.05 vs MS + T100 (n = 4/group). HO-1, heme oxygenase-1; Nrf-2, the nuclear factor erythroid 2-related factor 2; p-NF-kB, phosphorylated nuclear factor kappa B; PPARγ, peroxisome proliferator- activated receptor gamma; MS, metabolic syndrome; T100, tuna protein hydrolysate (100 mg/kg); T300, tuna protein hydrolysate (300 mg/kg); T500, tuna protein hydrolysate (500 mg/kg); Met100, metformin (100 mg/kg). Original blots are presented in Supplementary Fig. 11.

Down-regulation of HO-1 (Fig. 11a), Nrf-2 (Fig. 11b), and PPARγ (Fig. 11d) protein expression but up-regulation of p-NF-κB (Fig. 11c) protein expression were observed in MS rats compared to controls (*p* < 0.05). Treatment with TPH (300 or 500 mg/kg) or metformin for four weeks significantly reversed the alterations of these protein expressions when compared to rats (*p* < 0.05).

## Discussion

TPH used in the present study composes of protein, fat, ash and NaCl. TPH contains at least 20 amino acids, with glutamic acid and aspartic acid having the highest concentrations. Significant evidence backed up our findings that the primary component of fish protein hydrolysates is protein^32,33^. The primary determinant of the protein's nutritional value is its relative content of dietary necessary amino acids^34^. At many different biological levels, peptides and their bioactive amino acids have a nutritional and functional purpose^35^. The antioxidant and anti-inflammatory effects of TPH have been reported to associate with its amino acid sequences^36,37^.

Long-term consumption of a high caloric diet is a key factor that causes metabolic disorders. In a rat model of MS induced by a HFD, we showed alterations of metabolic parameters such as dyslipidemia, hyperglycemia, impaired OGTT, obesity, and hypertension. These results agreed with previous studies^3,4^. TPH supplementation alleviated metabolic disorders in a dose dependent manner. We selected TPH at a dose 300 mg/kg for the optimal or effective dose since most actions of TPH at either 300 or 500 mg/kg did not differ. A previous study in patients with type 2 diabetes mellitus showed improvement of glucose and lipid metabolism after supplementation with marine fish hydrolysates^38^. Metabolic disorders, especially high blood pressure, lead to cardiovascular complications. Reduced EF and FS implied the impairment of cardiac performance in MS rats that could be the consequence of LV hypertrophy. The echocardiogram showed decreased LVIDd and increased LVPWd that was consistent with the histological results of LV hypertrophy, increased wall thickness, increased luminal area, and CSA. These cardiac changes were resolved by TPH supplementation. It is known that cardiac hypertrophy is the compensatory response to pressure and/or volume overload in order to maintain cardiac output^39^. In addition, neurohumoral factors, including catecholamines and Ang II are important pathogeneses of cardiac hypertrophy^39^. Therefore, both the pressure overload (hypertension) and humoral factors (RAS activation) as well as insulin resistance that occurred in this rat model were suggested to induce LV morphological hypertrophy and then functional changes. One of the mechanisms responsible for anti-hypertensive and cardiac effects of TPH in the MS rats observed in this study may relate to its inhibitory capacity on ACE activity, which subsequently reduced systemic Ang II concentration and vascular tone. Emerging evidence supports the action of hydrolysates-derived from tuna that possess ACE inhibitor activity in vitro^31,40,41^. TPH also reduced the circulating leptin, which may subsequently have suppressed RAS activation in this observation.

The vascular responses to ACh were decreased while responses to SNP were not altered, indicating endothelial dysfunction in both the aorta and mesenteric vascular beds^42^. In addition, the responses to electrical field stimulation were augmented but the response to exogenous NE was unchanged. These results implied the enhancement of pre-junctional sites^9^. It is likely that vascular dysfunction is the major cause of hypertension in HFD rats. TPH improved vascular function in the present study, potentially through reducing oxidative stress in cardiovascular tissue and raising NO bioavailability in circulation. A previous study reported that protein hydrolysate of a tuna processing by-product enhanced NO production in human umbilical vein endothelial cells^31^. Furthermore, the increased NOx following TPH supplementation in this study might be owing to its ability to reduced oxidative stress since superoxide quickly reacts with NO to form peroxynitrite^43^. The enlargement of aortic walls and aortic hypertrophy also found in MS rats were reversed in rats treated with TPH. Generally, the process of aortic hypertrophy requires oxidative stress and inflammation^18^. The vascular effects of salmon protein hydrolysate have been noted as it decreases atherosclerosis in apolipoprotein (-/-) mice accompanied by reduced inflammation^30^. Several publications confirm the anti-oxidative and anti-inflammatory capacities of fish protein hydrolysates, especially from tuna^40,44^. TPH also reduced circulating TNF-α in the MS-treatment groups. Moreover, it could suggest that the antioxidant properties of TPH in this study involved at least two possible reasons: (1) TPH suppressed RAS with ACE inhibition and reduced the Ang II/AT1R/NOX2 signaling pathway and (2) TPH directly scavenged free radicals or enhanced Nrf2/HO-1 protein expression.

The molecular mechanisms of cardiac hypertrophy in MS rats were explored and an enhancement of AT_1_R/NOX2 protein expression was seen in cardiac tissue. It is well established that the AT_1_R/NOX2 signaling pathway mediates Ang II-induced cardiac hypertrophy^45,46^. Subsequently, growing evidence shows the crucial role of oxidative stress generated by activation of the AT_1_R/NOX2 signaling pathway, which contributes to the development of cardiac hypertrophy in rodents^47,48^. Our results supported the potent role of TPH in reducing LV hypertrophy via suppression of AT_1_R/NOX2 protein expression in cardiac tissue in the MS-treated groups. Reduced NO metabolite levels and endothelial dysfunction in the MS group were associated with decreased eNOS protein expression. Under normal physiological conditions, NO is synthesized by eNOS and induces relaxation of smooth muscle cells^49^. Therefore, in this study, TPH-improved endothelial function took place through the upregulation of eNOS protein. Oxidative stress in vascular tissue in the MS-only group was confirmed by the reduction of Nrf2/HO-1 expression, which was recovered by TPH supplementation. Activation of the Nrf2/HO-1 pathway plays an important role in increasing endogenous antioxidant enzymes to balance free radicals in cardiovascular complications^50^. Furthermore, aortic remodeling was associated with reduced PPARγ and increased NF-κB protein expression. It is known that activation of PPARγ can exert an anti-inflammatory effect via inhibiting the NF-κB signaling pathway and the inflammatory cytokine TNF-α^51^. Decreased expression of PPARγ in the coronary arterioles associated with vascular remodeling in the MS-only group has been reported. Furthermore, the link between oxidative stress and inflammation might involve the expression of PPARγ, since its expression was attenuated by oxidative stress in vascular endothelial cells. These results suggest that TPH decreased inflammation via modulation of the PPARγ/ NF-κB signaling pathway.

**Figure 12:**
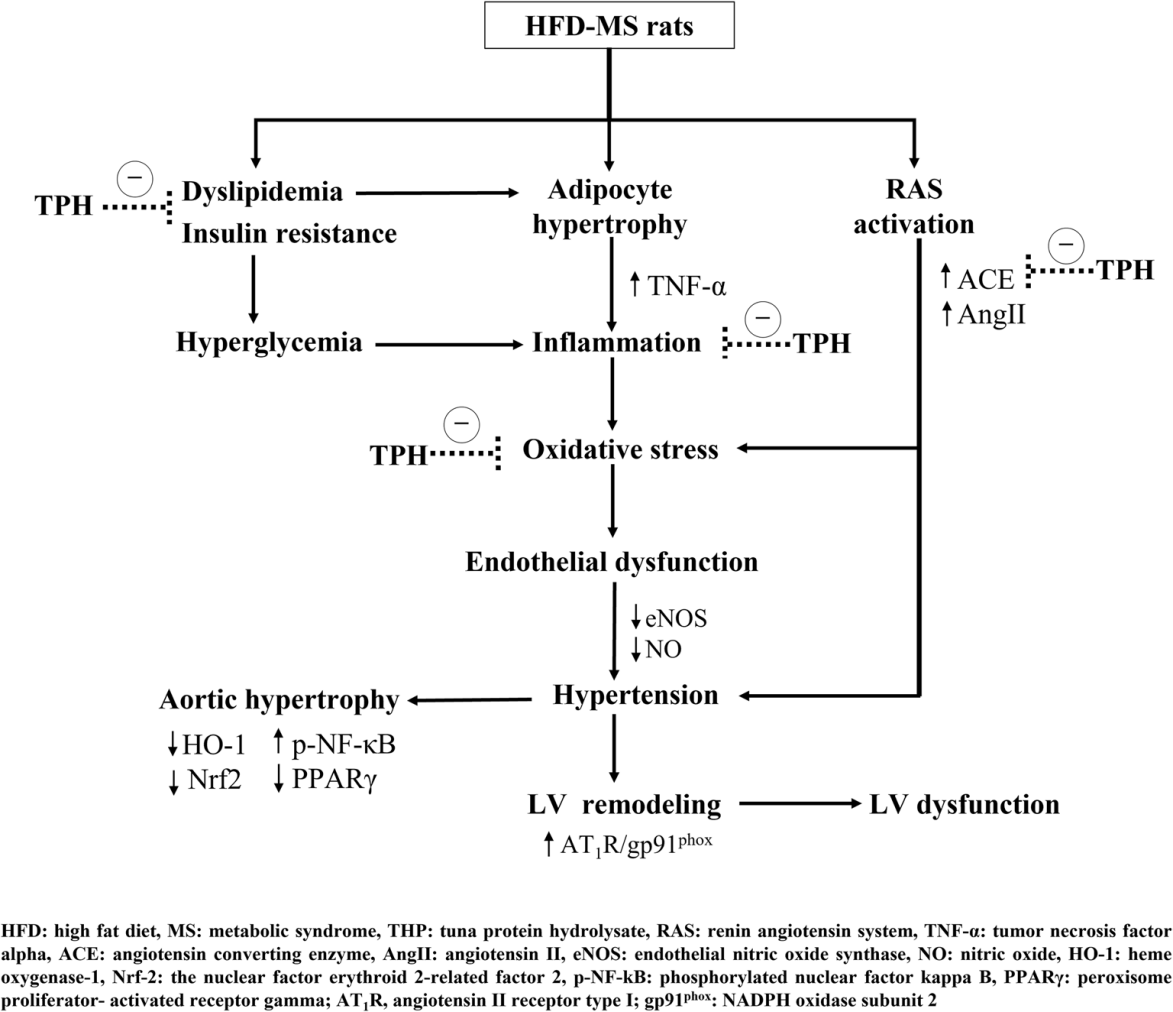
Proposed mechanisms of action of tuna protein hydrolysate.

A positive control group was formed using metformin. Similar to TPH, metformin has an attenuating effect on metabolic disorders and cardiovascular complications. Metformin is a standard drug used for the treatment type 2 diabetes through its ability to reduce hepatic gluconeogenesis and oppose the action of glucagon^52^. These results are supported by several publications that a metformin dose of 100 mg/kg can resolve MS in rats^53,54^. Furthermore, metformin can suppress Ang II-induced cardiovascular remodeling by reducing AT_1_R expression in cardiovascular cells^55^. The antioxidant and anti-inflammatory effects of metformin in the present study have been demonstrated by previous observations in fructose-fed streptozotocin-induced diabetic rats^56^. Metformin has also been shown to enhance PPARγ expression in MS rats^54^ and activate the Nrf2/HO-1 signaling pathway to alleviate oxidative stress in type 2 diabetic osteoporosis^57^. The graphical abstract of this study is demonstrated in Fig. 12. However, this study has some limitations, the information about the active ingredients and its quantities of TPH in rat serum was not provided.

In conclusion, TPH supplementation alleviated metabolic disorders and cardiovascular complications in MS rats. TPH suppressed RAS activation, oxidative stress, and inflammation. These effects were related to the modulation of AT_1_R/NOX2, eNOS, Nfr2/HO-1, and PPARγ/ NF-κB protein expression in cardiovascular tissue.

## Methods

### Chemicals

TPH was provided by Global Innovation Center (Thai Union Inc. Samutsakhon, Thailand). Metformin was obtained from Siam Pharmaceutical Company Ltd. (Bangkok, Thailand). All other chemicals used in this study were purchased from standard suppliers.

### Assays of TPH compositions and amino acid profiles

TPH was analyzed for the proximate compositions using the association of analytical chemists (AOAC) method. Amino acids profiles of TPH were analyzed by a high-performance liquid chromatography system.

### Animals and study protocols

Six-week-old male Sprague–Dawley rats (220–260 g), were purchased from Nomura Siam International Co., Ltd., Bangkok, Thailand. The animals were housed in a temperature-controlled environment (23 ± 2 °C). They were subjected to a 12/12 h light/dark cycle. All animal procedures were carried out in accordance with the ethical guidelines for the Care and Use of Laboratory Animals that are approved by the animal ethics committee of Khon Kaen University (IACUC-KKU-111/64), Thailand. This study is reported in accordance with Animal Research: Reporting of In Vivo Experiments (ARRIVE) guidelines.

The rats were divided into two groups: control (n = 8) and MS (n = 40). Control rats were fed a standard chow diet with 5.72 g fat/100 g, 22.9 g protein/100 g, and 57.81 g carbohydrates/100 g, while MS rats were fed a HFD with 24.29 g fat/100 g, 13.25 g protein/100 g, 46.3 g carbohydrates/100 g, and 15% fructose in drinking water (12 h during night-time). After a 12-week induction period, blood samples were collected via the lateral tail vein for FBG and lipid profile measurement to confirm MS in rats that received the HFD.

At 12 weeks, MS rats were subdivided into 5 groups (n = 8/group), MS rats + vehicle, MS rats + TPH (100, 300, or 500 mg/kg), and MS + metformin 100 mg/kg by oral gavage for the last 4 weeks.

### Indirect blood pressure measurement

Changes in SP were examined monthly in conscious rats using tail-cuff plethysmography with a Volume Pressure Recording sensor (CODA™, Kent Scientific corporation, Torrington, CT, USA).

### Measurements of body weight and metabolic parameters

Each rats' body weight (BW) was measured once a week. Rats were overnight fasted at weeks 12 and 16 for the measurement of FBG with a glucometer (Roche Diagnostics Australia Pty. Ltd., Sydney, Australia). To represent glucose tolerance in rats, the OGTT and the AUC derived from the OGTT were performed. Briefly, the rats were given a 20% glucose solution (2 g/kg BW) orally via a gastric tube. Blood samples were taken from the lateral tail veins at different time intervals (0, 30, 60, 120, and 180 min) after glucose feeding.

### Echocardiogram procedures

At the end of the experiment, rats were anesthetized by thiopental sodium (50 mg/kg). The protocol is given in previous studies^8,58^. Echocardiograms were performed using the Veterinary Diagnostic Ultrasound system Vetus 8 (Shenzhen Mindray Animal Medical Technology Co. Ltd, Shenzhen, China). LV structure and function were assessed using the two-dimensional short-axis view. Subsequently, M-mode tracings were recorded as follows: for LVIDd **and** LV internal dimension at end-systole **(**LVIDs**)**; interventricular septum at diastole (IVSd) and at systole (IVSs); LVPWd and at systole (LVPWs); and EDV, end systolic volume (ESV), and SV from three consecutive cardiac cycle. % FS was calculated from the following equation: % FS = [(LVIDd-LVIDs)/LVIDd] × 100.

### Hemodynamic parameter measurement

The inguinal region of rats was surgically prepared. The left femoral artery was cannulated with a polyethylene tube connected with the Acknowledge Data Acquisition software (Biopac Systems Ins., Santa Barbara, CA, USA). Baseline values of SP, DP, MAP, PP and HR were continuously monitored for 30 min. At the end of the experiment, rats were scarified by exsanguination and blood was collected via the abdominal aorta. Heart and epididymal fat (EP) weight were measured.

### Assay of lipid profiles, plasma leptin level, TNF-α, and liver function

Blood samples were immediately separated by centrifugation at 3,500 rpm, at 4 °C for 30 min. TC, TG, and HDL-c levels in plasma were determined spectrophotometrically using specific commercial kits (Human Gesellschaft fuer Biochemica and Diagnostica mbH, Wiesbaden, Germany). Leptin levels in plasma were determined using commercial kits (RAB0335, Sigma-Aldrich, St. Louis, MO, USA). Plasma TNF-α was assayed using commercial kits (RAB0479, Sigma-Aldrich, Saint Louis, MO, USA). Liver function was determined using AST (MAK055-1KT) and ALT (MAK052-1KT) activities in serum (Sigma-Aldrich, St. Louis, MO, USA). All protocols followed the manufacturers’ instructions.

### Biochemical measurements

#### Assay of plasma nitric oxide metabolite concentrations

NOx concentration was examined using an enzymatic conversion method followed by reaction with a Griess reagent. The absorbance of dilution was measured on an ELISA plate reader with a filter wavelength of 540 nm (Tecan GmbH., Grodig, Australia). NaNO_2_ reacted with a Griess substance to produce a standard curve^59^. The value of NOx was expressed as µM.

#### Assay of plasma Ang II level

The concentration of plasma Ang II was measured using an Ang II Enzyme immunoassay (EIA) kit (RAB0010-1KT, St. Louis, MO, USA) according to the manufacturer’s instructions.

#### Assay of ACE activity

Serum ACE activity was measured using a fluorescence assay following a previously described method^60^ with some modifications. Briefly, 25 μL of serum was mixed with 15 mM hippuryl-L-histidyl-L-leucine (HHL) in an assay buffer containing 20 mM sodium borate and 0.3 M NaCl, with a pH of 8.3 at a final volume of 125 μL. The mixtures were incubated at 37 °C for 30 min, and the reaction was stopped by adding 150 μL of 0.1 M NaOH. The product of the reaction was labeled with 10 mg/mL o-phthaldialdehyde (OPA) and read on a microplate reader at 390 nm. ACE activity was reported as mU/mL.

### Vascular function study

#### Experimental protocols in isolated mesenteric vascular beds

The mesenteric vascular bed (MVB) was carefully isolated and placed on a stainless-steel grid in a humid chamber to maintain temperature. Preparations were perfused using physiological Krebs’ solution at a constant flow rate of 5 ml/min. After 30 min of acclimatization, the contractile response to an EFS at 5–40 Hz, 90 V, 1 ms, for 30 s with 5 min intervals was performed. Changes in the mean perfusion pressure (mmHg) were detected using a BIOPAC System (BIOPAC System Inc., CA, USA). Thereafter, the contractile response to exogenous NE was determined by injection of a bolus dose of NE from 0.15–15 nmol into the MVB. In addition, methoxamine (5–7 µM) was added into Krebs’ solution to increase the tone for evaluating the vasoactive performance of resistance in small arteries. Subsequently, different doses of the vasoactive agents, ACh (1 nM-0.01 µM) or SNP (1 nM–0.01 µM) were injected through a neoprene rubber tube into the tissue.

#### Experimental protocols in isolated aortic rings

The thoracic aorta was quickly removed, cleaned, and cut into 1 cm length pieces for tension measurement. The rings were incubated for 1 h in Krebs' solution baths (37 ° C with a 95% O_2_ and 5% CO_2_ gas mixture). Changes in the tension of the rings were measured using a transducer connected to a 4-channel bridge amplifier and PowerLab A/D converter as well as a PC running Chart v.5 (PowerLab System. AD Instrument, Australia). After the equilibration period, the ring was toned with phenylephrine (10 µM) and the vascular responses to ACh (0.01–3 µM) or SNP (0.01–3 µM) were measured, respectively.

### Measurement of oxidative stress markers

#### ***Assay of vascular O***_***2***_^***•–***^*** production and malondialdehyde (MDA) levels***

The production of O_2_^•–^ in the aortic tissue and the heart samples of all rats were determined based on the lucigenin-enhanced chemiluminescence method as described previously^61^. The production of O_2_^•–^ was exhibited as relative light unit counts/minute/dried weight of aorta or heart. The levels of MDA in the tissues (aortic and cardiac tissue) were determined following the previously described protocol^58,62,63^. Data were expressed as µmol/g of protein and µmol/mL plasma, respectively.

#### Assays tissue CAT activities

The levels of CAT activity in the aorta and cardiac tissue were measured using the method described in the previous publication^64^. In brief, the supernatant or standard solution was added to the microplate before 50 µL of 30% H_2_O_2_ in 50 nM potassium phosphate buffer (pH 7.0) was dripped in. Afterward, 25 µL of 5N H_2_SO_4_ was added and mixed thoroughly before adding 150 µL of KMnO_4_ to stop the reaction. The activity was measured at 490 nm. The data were expressed as U/mg protein.

### Histological and morphometric analysis

The samples of cardiac, epididymal fat, and aortic tissue were fixed in 10% formalin for 48 h before being dehydrated through a serial alcohol solution. These tissues were embedded in paraffin before being cut into 5 μm thick sections. To determine structural changes, all sections were stained with H&E (Bio-Optica Milano SpA, San Faustino, Milano, Italy). The slides were examined with an eclipse Ni-U upright microscope (Nikon, Tokyo, Japan) and analyzed with ImageJ software (National Institutes of Health, Bethesda, MD, USA). Previous studies' morphometric analysis protocols were followed for each tissue^8,65^.

### Protein expression by western blotting

Western blotting was used to assess protein expression in the heart and aorta. AT_1_R and gp91^phox^ protein expression levels were measured in cardiac tissue, while eNOS, HO-1, Nrf-2, PPAR-γ, and p-NF-_κ_B protein expression were measured in aortic tissue. After homogenizing the tissue samples, the extracted proteins (20–50 mg of protein) were separated and electrophoretically transferred to a polyvinylidene difluoride membrane. At room temperature, the membranes were blocked for 2 h with 5% BSA in TBS with 0.1% Tween20. Subsequently, an overnight incubation at 4 °C with mouse monoclonal antibodies to AT_1_R **(**sc-515884, 1:500**)**, gp91^phox^ **(**sc-74514, 1:500**)**, PPARγ **(**sc-27139, 1:1000**)**, HO-1 **(**sc-136960, 1:500**)**, Nrf-2 **(**sc-365949, 1:500**)** (Santa Cruz Biotechnology, Inc., Dallas, Texas, USA), eNOS [**(**610296**)**, 1:250, BD Transduction Laboratories™, CA, USA], and rabbit monoclonal antibodies to p-NF-kB **(**3033S, 1:1000**)** (Cell signaling, Theera Trading Co. Ltd., Bangkok, Thailand) was performed. Following the incubation period, the membrane was washed three times with TBST and then incubated for 1 h at room temperature with horseradish peroxidase conjugated secondary antibody. The signals were developed in Immobilon Forte Western HRP Substrate (EMD Millipore Corp., Burlington, MA, USA) and detected with an Amersham Imager 600 (GE Healthcare Life Science, Uppsala, Sweden). These protein band intensities were normalized to β-actin. The results were expressed as percentages of the values compared to the control group from the same gel.

### Statistical analysis

Data are presented as mean ± the standard error of the mean (SEM.). One-way analysis of variance (ANOVA) was used for statistical analysis, followed by the Tukey post hoc test. Statistical significance was determined at a p-value < 0.05. GraphPad Prism software Inc. (San Diego, CA, USA) was used for statistical analysis.

## Supplementary Information

Supplementary Figures.

## Data Availability

Correspondence and requests for materials should be addressed to Poungrat Pakdeechote.
