# Supplementary material for: Cardiovascular complications are resolved by tuna protein hydrolysate supplementation in rats fed with a high-fat diet
Source: Sci Rep. 2023 Jul 28;13:12280. doi: 10.1038/s41598-023-39538-z (PMC10382531; doi:10.1038/s41598-023-39538-z)
Supplement: Supplementary file 1 — Supplementary Figures. [file 41598_2023_39538_MOESM1_ESM.pdf]

Supplementary information

Cardiovascular complications are resolved by tuna protein hydrolysate supplementation in rats fed with a high-fat diet

Putcharawipa Maneesai[1], Jintanaporn Wattanathorn[1,3], Prapassorn Potue[1], Juthamas Khamseekaew, Siwayu Rattanakanokchai, Wipawee Thukham-Mee[1,3], Supaporn Muchimapura[1,3], Poungrat Pakdeechote[1*]

[1] Department of Physiology, Faculty of Medicine, Khon Kaen University, Khon Kaen, 40002, Thailand

[2] Research Institute for Human High Performance and Health Promotion, Khon Kaen University, Khon Kaen, 40002, Thailand

[3] Faculty of Veterinary Medicine, Khon Kaen University, Khon Kaen 40002, Thailand

[*] Corresponding author: ppoung@kku.ac.th

S1: Western blots of three independent experiments showing the respective target bands of the A) Angiotensin II receptor type I (AT_1R) at 43 kDa and B) NADPH oxidase subunit 2 ($gp91^{phox}$) at 60 kDa in cardiac tissue which normalized to beta actin at 42 kDa. Control, normotensive rats; MS, metabolic syndrome rats; MS + T100, metabolic syndrome rats treated with 100 mg/kg tuna-derived protein hydrolysate; MS + T300, metabolic syndrome rats treated with 300 mg/kg tuna-derived protein hydrolysate; MS + T500, metabolic syndrome rats treated with 500 mg/kg tuna-derived protein hydrolysate; MS + Met100, metabolic syndrome rats treated with 100 mg/kg metformin.

Figure 9A

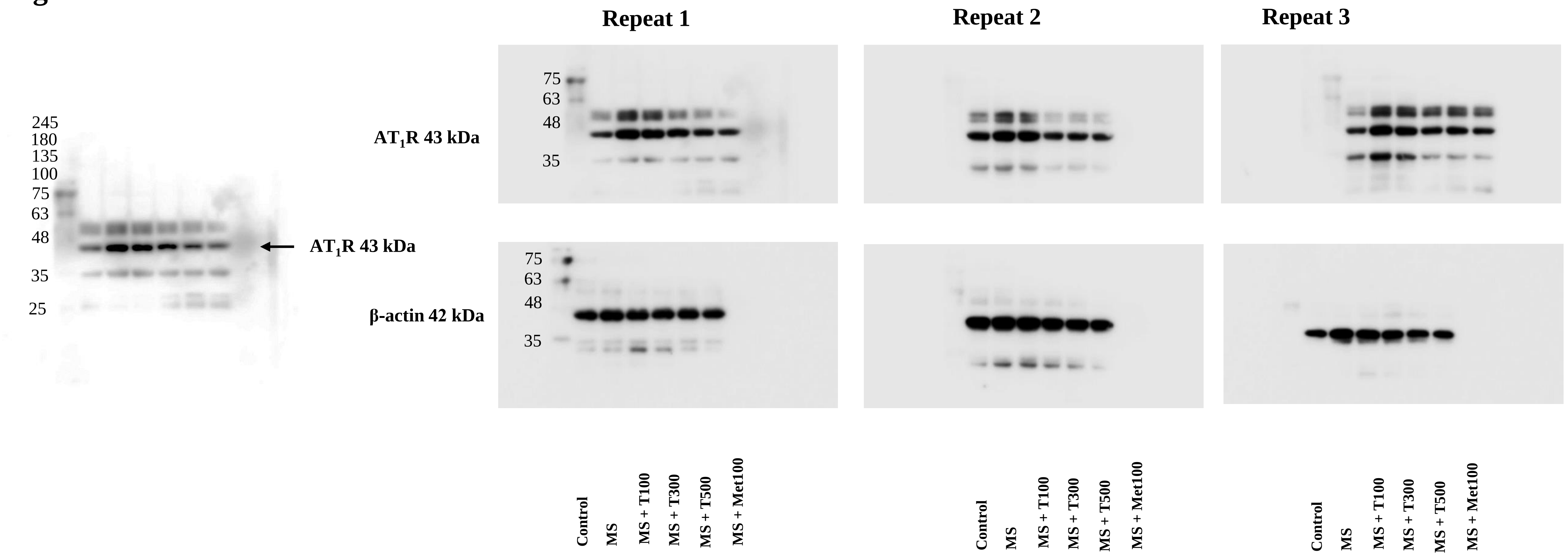

Figure 9B

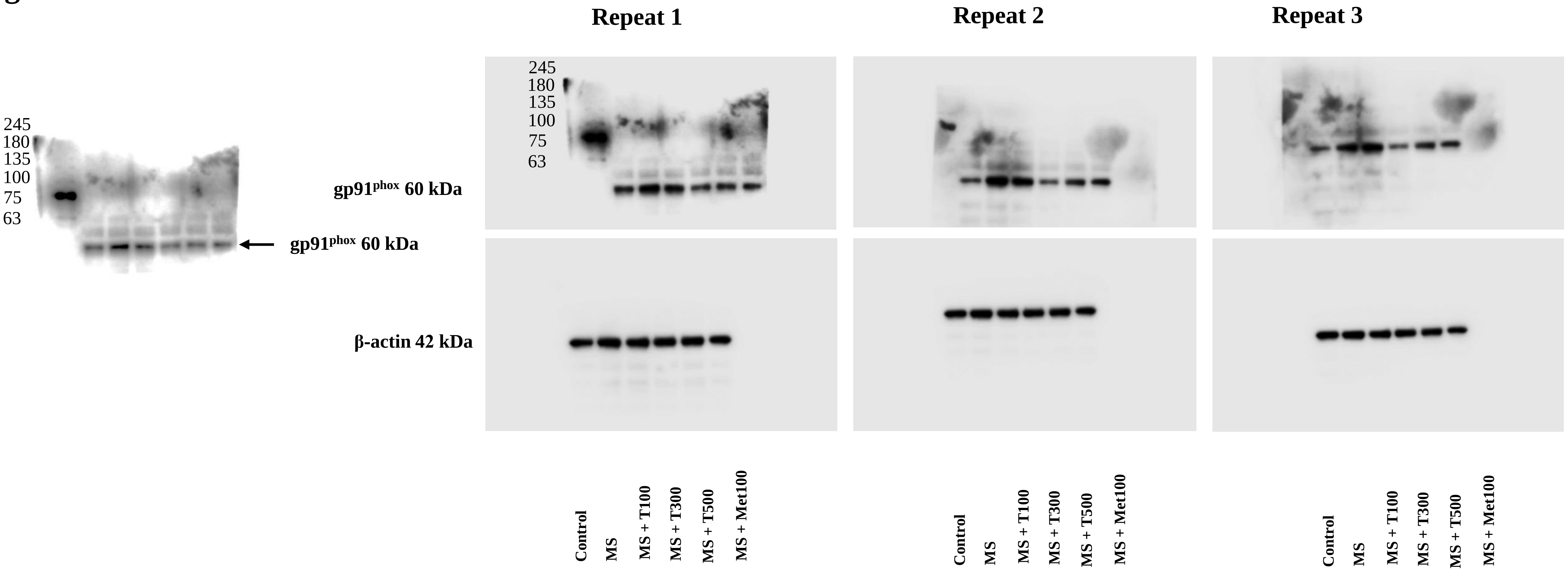

Repeat 1
Repeat 2
Repeat 3
245
180
135
100
75
63
gp91phox 60 kDa
gp91phox 60 kDa
β-actin 42 kDa
Control
MS
MS + T100
MS + T300
MS + T500
MS + Met100

S2: Western blots of three independent experiments showing the respective target bands of the A) endothelial nitric oxide synthase (eNOS) at 140 kDa in aortic tissue which normalized to beta actin at 42 kDa. Control, normotensive rats; MS, metabolic syndrome rats; MS + T100, metabolic syndrome rats treated with 100 mg/kg tuna-derived protein hydrolysate; MS + T300, metabolic syndrome rats treated with 300 mg/kg tuna-derived protein hydrolysate; MS + T500, metabolic syndrome rats treated with 500 mg/kg tuna-derived protein hydrolysate; MS + Met100, metabolic syndrome rats treated with 100 mg/kg metformin.

Figure 10

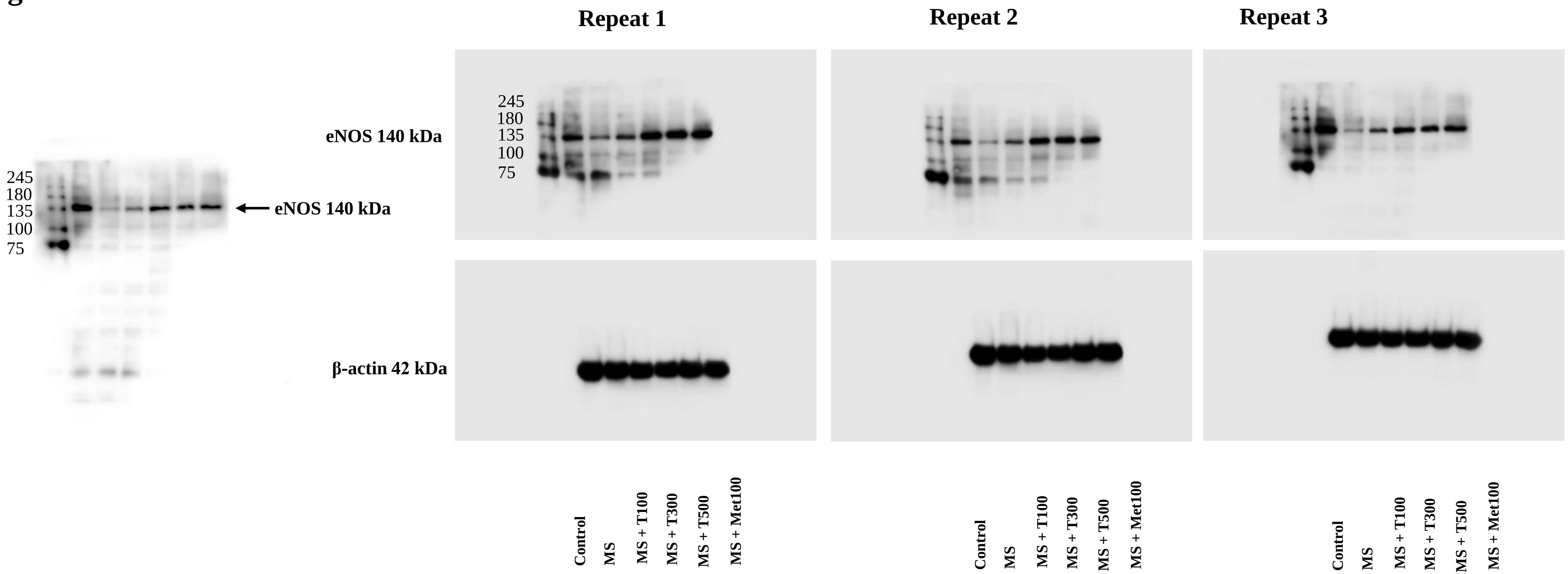

S3: Western blots of three independent experiments showing the respective target bands of the A) heme oxygenase-1 (HO-1) at 32 kDa, B) the nuclear factor erythroid 2-related factor (Nrf2) at 61 kDa, C) phosphorylated nuclear factor kappa B (p-NF-kB) at 65 kDa and D) peroxisome proliferator- activated receptor gamma (PPARγ) at 54 kDa in aortic tissue which normalized to beta actin at 42 kDa. Control, normotensive rats; MS, metabolic syndrome rats; MS + T100, metabolic syndrome rats treated with 100 mg/kg tuna-derived protein hydrolysate; MS + T300, metabolic syndrome rats treated with 300 mg/kg tuna-derived protein hydrolysate; MS + T500, metabolic syndrome rats treated with 500 mg/kg tuna-derived protein hydrolysate; MS + Met100, metabolic syndrome rats treated with 100 mg/kg metformin.

Figure 11A

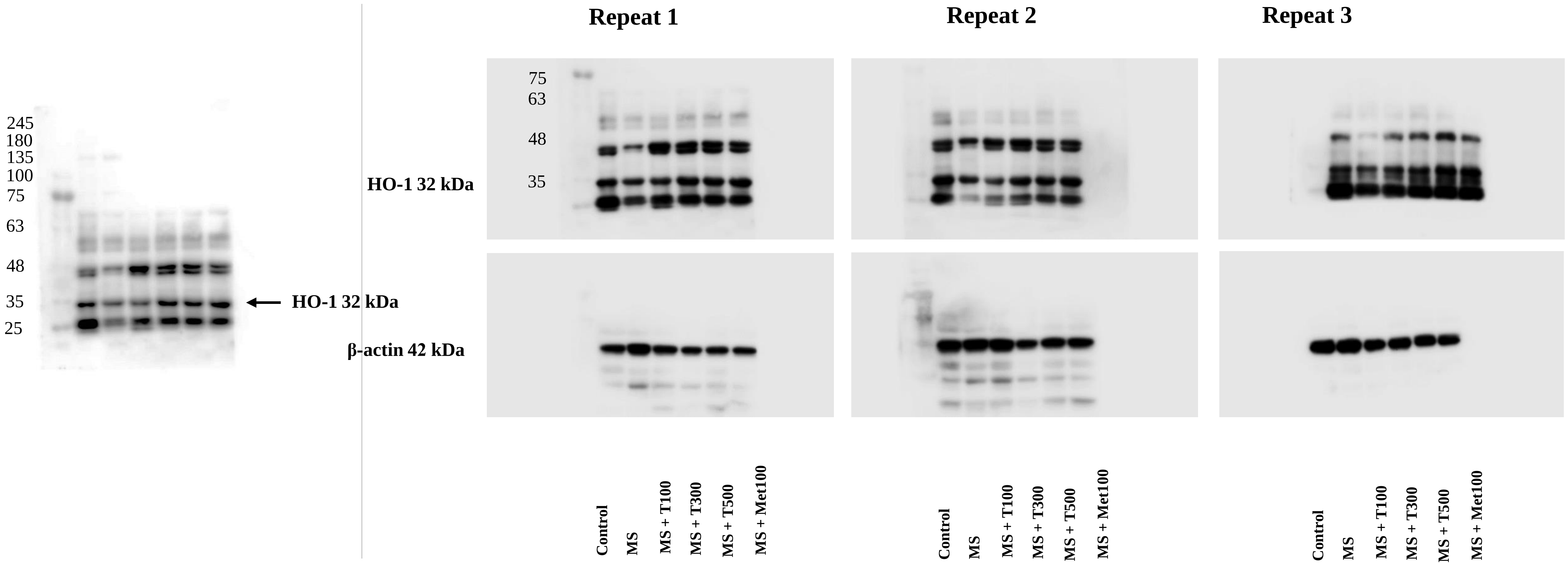

Figure 11B

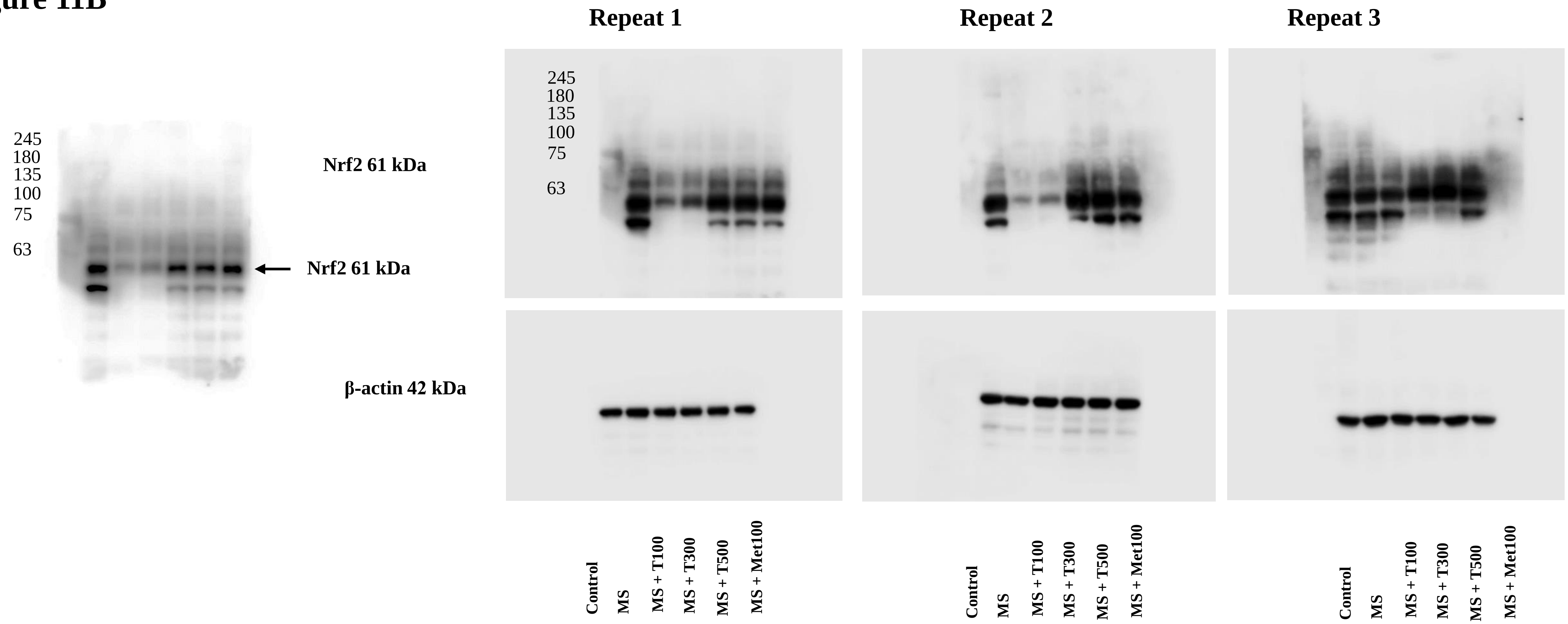

Repeat 1
Repeat 2
Repeat 3
245
180
135
100
75
63
Nrf2 61 kDa
β-actin 42 kDa
Control
MS
MS + T100
MS + T300
MS + T500
MS + Met100

Figure 11C

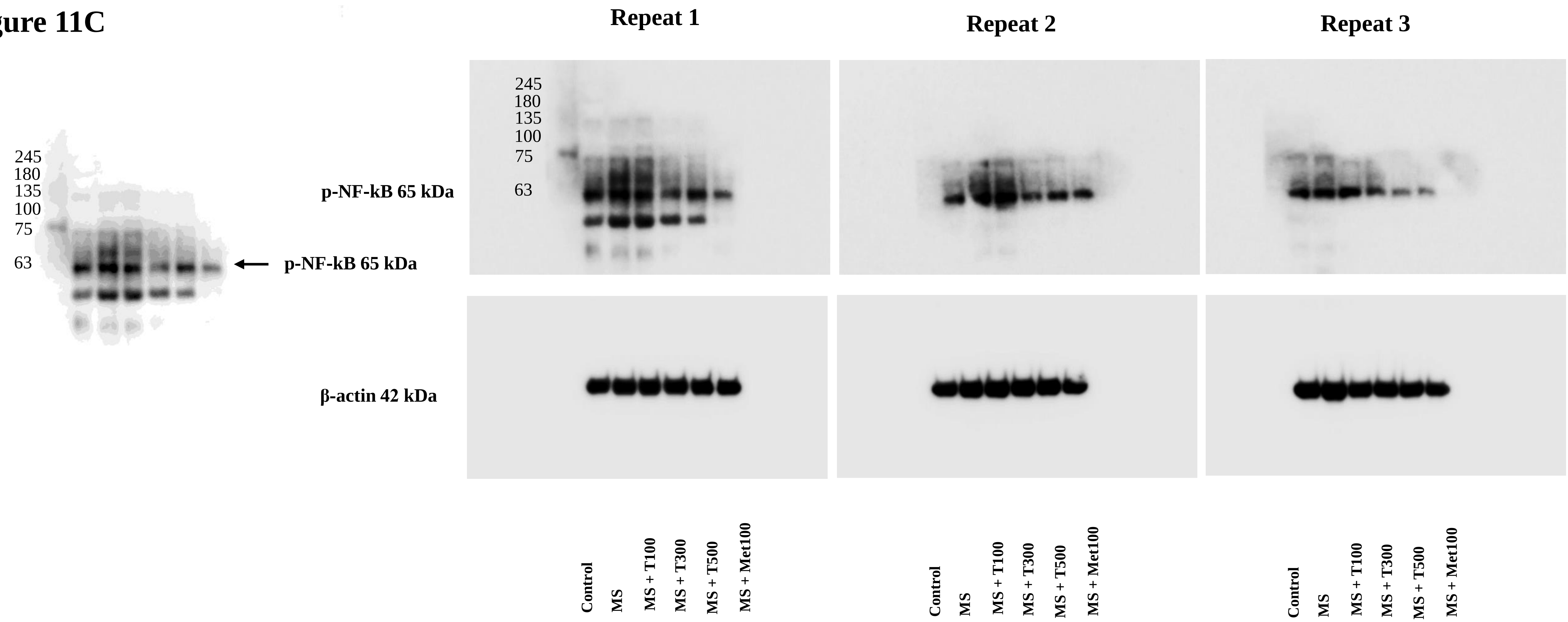

Repeat 1
Repeat 2
Repeat 3
245
180
135
100
75
63
p-NF-kB 65 kDa
p-NF-kB 65 kDa
β-actin 42 kDa
Control
MS
MS + T100
MS + T300
MS + T500
MS + Met100

Figure 11D

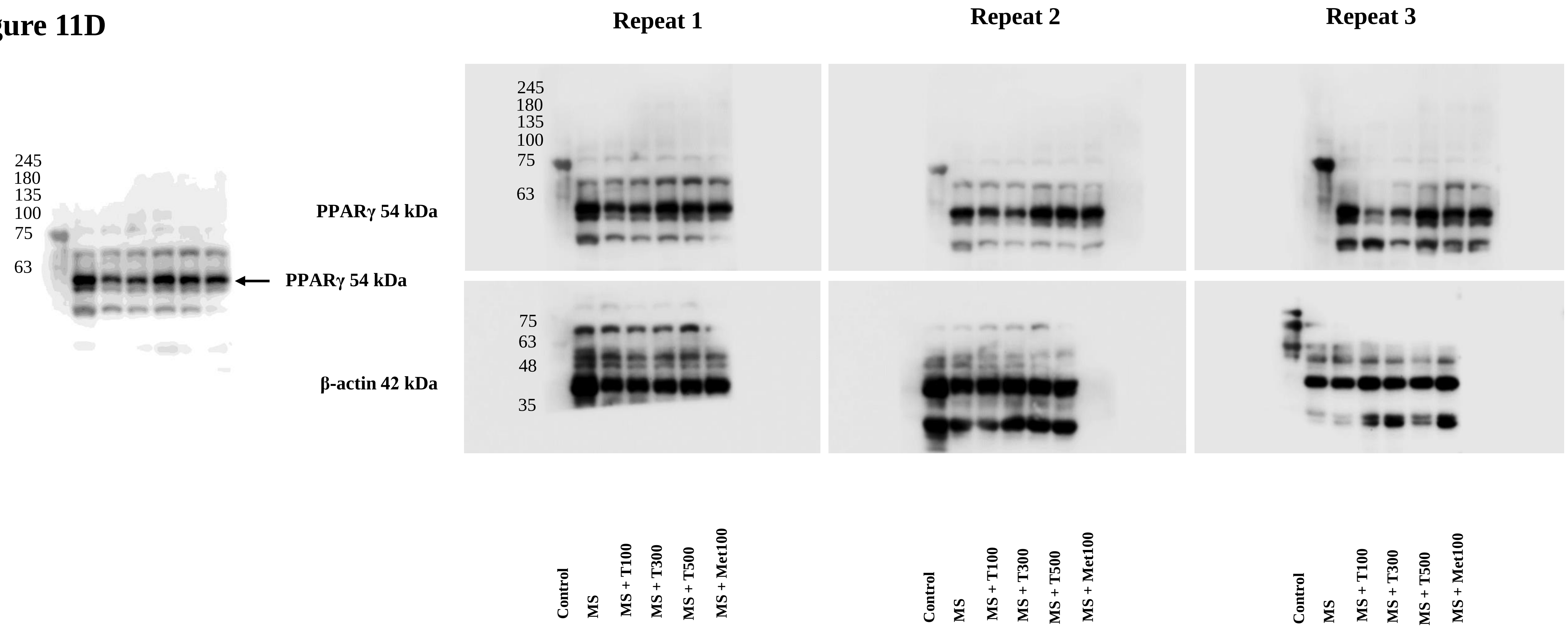

Repeat 1
Repeat 2
Repeat 3
245
180
135
100
75
63
PPARγ 54 kDa
PPARγ 54 kDa
β-actin 42 kDa
48
35
Control
MS
MS + T100
MS + T300
MS + T500
MS + Met100
